# Blood Biochemistry Analysis to Detect Smoking Status and Quantify Accelerated Aging in Smokers

**DOI:** 10.1038/s41598-018-35704-w

**Published:** 2019-01-15

**Authors:** Polina Mamoshina, Kirill Kochetov, Franco Cortese, Anna Kovalchuk, Alexander Aliper, Eugene Lane, Morten Scheibye-Knudsen, Charles R. Cantor, Neil M. Skjodt, Olga Kovalchuk, Alex Zhavoronkov

**Affiliations:** 1 Pharmaceutical Artificial Intelligence Department, Insilico Medicine, Inc., 9601 Medical Center Dr, Suite 127, JHU, Rockville, MD 20850 USA; 2 Canada Cancer and Aging Research Laboratories, Ltd, Lethbridge, Alberta T1K7X8 Canada; 3 Computer Science Department, University of Oxford, Oxford, United Kingdom; 4 Computer Technologies Lab, ITMO University, St. Petersburg, 197101 Russia; 5 Biogerontology Research Foundation, Research Department, Oxford, United Kingdom; 6 Canadian Longevity Alliance, Ontario, Canada; 7 University of Lethbridge, Lethbridge, Alberta T1K3M4 Canada; 8 Leaders in Medicine Program, Cumming School of Medicine, University of Calgary, Calgary, Alberta T2N 4N1 Canada; 9 Center for Healthy Aging, Department of Cellular and Molecular Medicine, University of Copenhagen, Copenhagen, Denmark; 10 Boston University, Department of Biomedical Engineering, Boston, Massachusetts 02215 USA; 11 Buck Institute for Research on Aging, 8001 Redwood Boulevard, Novato, CA 94945 USA

**Keywords:** Computational platforms and environments, Data processing, Health policy, Molecular medicine

## Abstract

There is an association between smoking and cancer, cardiovascular disease and all-cause mortality. However, currently, there are no affordable and informative tests for assessing the effects of smoking on the rate of biological aging. In this study we demonstrate for the first time that smoking status can be predicted using blood biochemistry and cell count results andthe recent advances in artificial intelligence (AI). By employing age-prediction models developed using supervised deep learning techniques, we found that smokers exhibited higher aging rates than nonsmokers, regardless of their cholesterol ratios and fasting glucose levels. We further used those models to quantify the acceleration of biological aging due to tobacco use. Female smokers were predicted to be twice as old as their chronological age compared to nonsmokers, whereas male smokers were predicted to be one and a half times as old as their chronological age compared to nonsmokers. Our findings suggest that deep learning analysis of routine blood tests could complement or even replace the current error-prone method of self-reporting of smoking status and could be expanded to assess the effect of other lifestyle and environmental factors on aging.

## Introduction

The population of nearly every nation is rapidly aging, a demographic trend that is expected to strain health care and social welfare programs^1^. A variety of biomarkers,quantitative physiological indicators of health status, can be used to assess individual biological aging rates and health risks^2,3^. These metrics aid the diagnosis and prognosis of diseases associated with aging, such as cancer^4^ and genetic diseases that result in premature aging^5^. Biomarker analysis may enable a quantitative assessment of the effect of environmental factors on the rate of biological aging, and may provide tools for evaluating the effect of promising anti-aging therapies in humans^6,7^.

Blood tests are a routine part of individual health assessment and serve as sensitive indicators for many diseases. The rapid accumulation of laboratory tests in public repositories is conducive to big data analysis. Sophisticated machine learning and deep learning techniques can surpass traditional statistical methods for handling large, complex, nonlinear, and multidimensional datasets^8–13^. We have shown previously that the hematological aging clocks built using feed-forward deep neural networks can be used to track age-related changes^12^ and can serve as better predictors of all-cause mortality than chronological age^13^. Such biomarkers can be used to study accelerated aging caused by hazardous environmental exposures.

One environmental factor in particular— tobacco smoking— exerts tremendous pressure on health-care systems worldwide causing death, morbidity, and possibly premature aging^14^. Previous studies demonstrated that smoking is strongly associated accelerated ageing indices such as the Frailty Index^15^ and mortality risk^16^. Lei *et al*.^17^ showed that tobacco smokers were predicted to be older while smoking and to be younger after smoking cessation as measured by DNA methylation clocks^17^.

To the best of our knowledge, this study offers the first large-scale deep learning-based analysis of clinical blood tests to assess predicting smoking status and the effect of tobacco smoking on the rate of biological aging. Our hematological aging clock complements the exciting models for age-prediction, and the smoking status predictor demonstrates the ability to assess the smoking status using blood biochemistry and cell count profiles.

## Results

**Figure 1:**
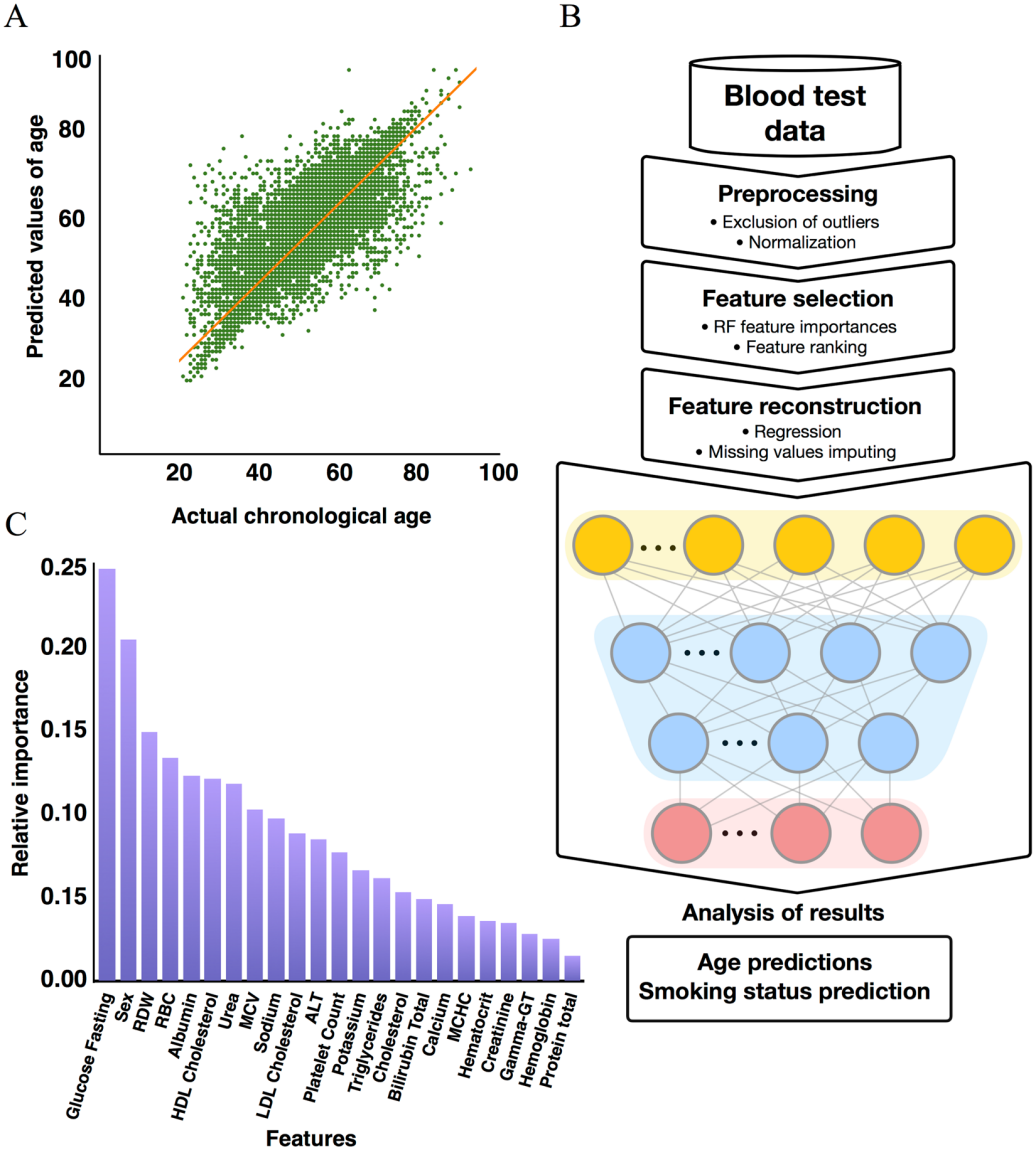
Deep learning-based blood-biochemistry clocks accurately predict chronological age. (**A**) Prediction accuracy of the best-performing model. The model trained on 24 parameters achieved an *R*^2^ of 0.57 and an *MAE* of 5.7 years. (**B**) The design of the deep learning study that used blood-biochemistry data to predict an individual’s age. Blood samples of nonsmokers were first preprocessed and normalized as previously described^8^. Next, arbitrage ranking based on 320 RF models was applied to facilitate the selection of the most appropriate feature space with maximum samples available. Afterward, missing values were reconstructed using an autoregressive model with a view towards increasing the training sets, and the resulting feature sets were used to train and test DNNs for predicting patient age and smoking status. (**C**) Feature importance plot. Fasting glucose, sex, and RDW exhibited higher relative importance scores than other features used in model training. *Note* High-density lipoprotein (HDL) cholesterol, low-density lipoprotein (LDL) cholesterol. RDW for red blood cell distribution width, RBC for red blood cell counts, MCV for mean corpuscular volume, ALT for alanine transaminase, MCHC for mean corpuscular hemoglobin.

To perform this study, we received a large administrative dataset of anonymized blood biochemistry and cell count results linked to individuals’ chronological age, sex, and confirmed smoking status. The dataset was representative of the entire Alberta population, both rural and urban, with proportional representation of individuals of all ethnic origins. We then trained a set of supervised feed-forward deep neural networks (DNNs) on the nonsmokers to predict the chronological age (Fig. 1B). Subsequently, we calculated the age of the smokers and nonsmokers excluded from the training. To further investigate the effect of smoking on age prediction, we included smoking status as one of the input features and performed feature importance (FI) analysis. Finally, we trained a set of supervised feed-forward deep neural networks to predict the smoking status of patients using only their blood profiles and sex.

### Data overview and preprocessing

We obtained data from 149,000 fully anonymized individual records linked to smoking status (49,000 smokers), sex, and age, with up to 66 blood biochemistry and cell count markers (Supp. Table 1). Of the 66 markers, 36 were among the 41 features used to train our previous Aging.AI 1.0 system^10^. The number of females, males, smokers and non-smokers within each age group was comparable (Supplementary Fig. 1). The median age was 55 years.

DNNs require large training datasets. To obtain a sufficiently large training sets we first selected samples with the same blood test date, that is, datasets consisting exclusively of blood-based biomarkers measured on the same day, so that our DNN could be trained consistently, relevantly, and accurately.

Although deep learning models can automatically extract features from the data and usually outperform shallow machine learning at this task, it is a good practice to select a set of relevant features before training the network. We optimized the feature spaces that were used to train the models for age prediction first excluding smoking status using a multifactorial adaptive statistical arbitrage model^13^ for subsets of samples with various numbers of measured markers. We trained 320 random forest (RF) models on distinct feature spaces and subsequently extracted FI values from each model. The features were ranked by their relative importance to age prediction according to the scores of the models (Formula 1, Supplementary Fig. 2). The accuracy of any predictor depends on the sample size and the feature space on which it is trained. To supplement the number of features used to train our predictors, we applied linear regression to fill missing values for 30–60% (depending on the feature type) of the samples in the dataset. This reconstruction successfully increased the number of available features from 14, 15, and 18 to 18, 20, and 23 features, respectively.

The blood marker with the largest contribution to the age-prediction model is glycated hemoglobin (hemoglobin A1c), followed in descending order by blood urea, fasting serum glucose, and serum ferritin (Supplementary Fig. 2). Fasting glucose was among the most important features in our previous studies on deep learning-based hematological aging clocks^10,11^.

Interestingly, the most important markers (as selected by the arbitrage FI method) demonstrate independent weak biweight mid-correlation, which shows the strength of a linear association between blood markers and age. The arbitrage FI method is more robust than the Pearson correlation coefficient, being a median-based measure that is less sensitive to outliers (Supplementary Fig. 3, Table 2).

### Deep-learned blood-biochemistry clocks can effectively predict biological age

Using the FI ranking determined by the RF models, we selected three different sets of blood biochemistry and cell count markers (Supplementary Table 3). Input feature sets were chosen to contain the maximum number of available samples that displayed the features selected via RF-based arbitrage feature selection previous section).

To predict individual age, we trained three DNNS on selected blood test input features of nonsmoking subjects. The predictive performance of each model was evaluated using the Pearson correlation coefficient (*r*), the standard coefficient of determination (*R*^2^), and the mean absolute error (*MAE*) (Formulae 2–4).

All three models achieved a relatively high correlation between predicted and actual chronological age. The best-performing model was the deep neural network trained on 23 blood test input features (*MAE* = 5.72 years, *R*^2^ = 0.56). The deep neural network trained on 20 blood test input features achieved an *MAE* of 5.78 years and an *R*^2^ of 0.578, followed by the deep neural network trained on the 18 available blood test input features, which achieved an *MAE* of 5.898 years and an *R*^2^ of 0.55 (Fig. 1A, Supplementary Fig. 4A,B, Table 1). Samples from the tail ends of the distribution (individuals younger than 35 years and those older than 75 years) exhibited a higher error rate for age prediction. Fasting glucose, sex, and red blood cell distribution width (RDW) were predicted to be the most important markers (Fig. 1C, Supplementary Fig. 4C,D).

### Deep-learned biochemistry clocks reveal differences in the biological ages of smokers and nonsmokers

**Figure 2:**
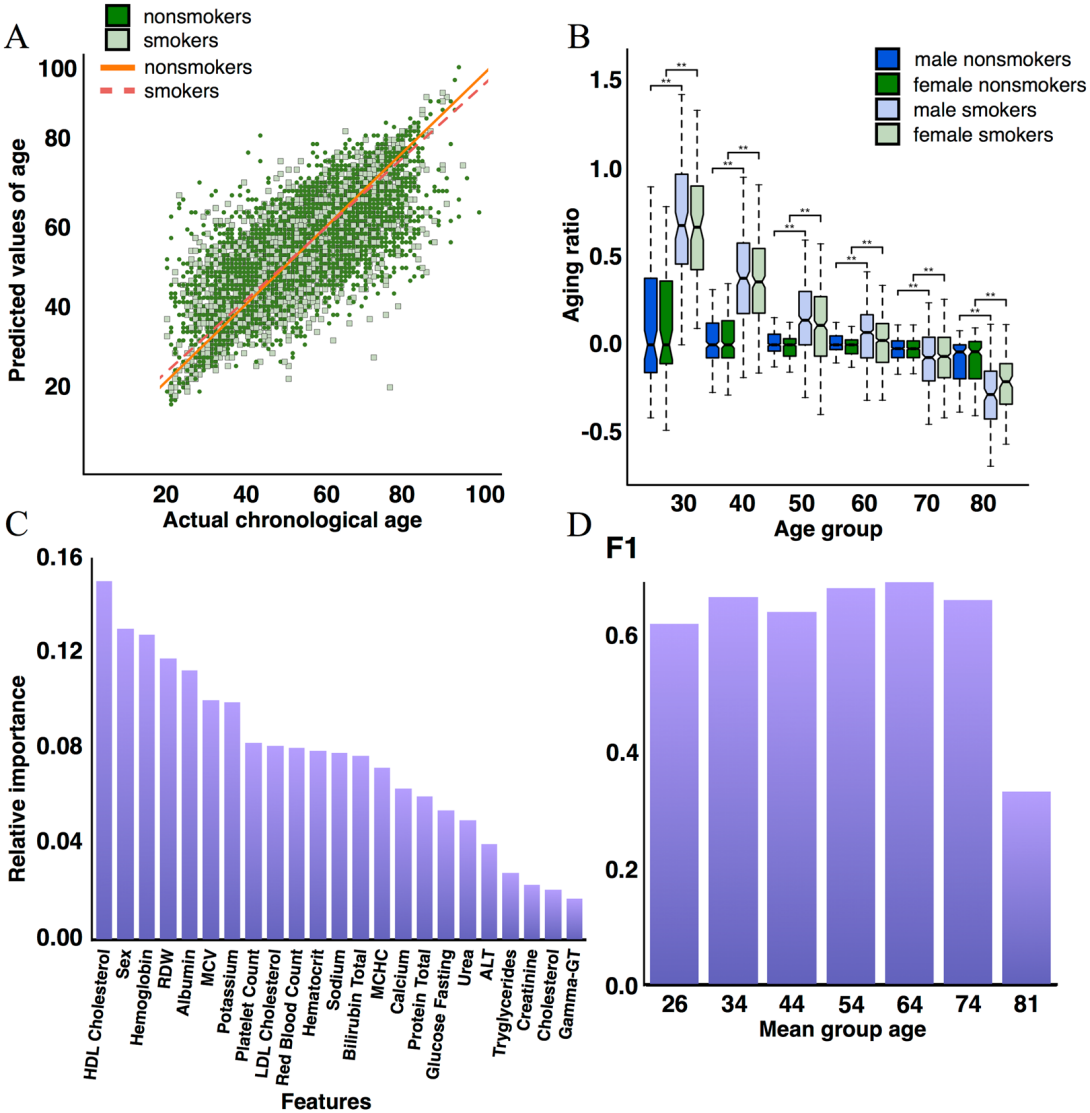
Deep learning-based hematological clocks demonstrated accelerated aging rates in smokers and revealed patient smoking status. (**A**) The prediction accuracy of the best-performing model trained on feature space extended with smoking status. The model, trained on 24 parameters, achieved an *R*^2^ of 0.60 and an *MAE* of 5.42 years (**B**) The log_2_ aging ratio of smokers to nonsmokers by age and sex groups for the best-performing model. Smokers demonstrated a higher aging rate regardless of sex. However, these differences plateaued after 55 years of age. A log_2_ aging ratio of 1 means the sample was predicted to be twice as old as a chronological age, and a log_2_ aging ratio of −1 means the sample was predicted to be half as old as a chronological age. (**C**) The most important features in the classification of smoking status selected by the PFI method. HDL cholesterol, sex, and hemoglobin exhibited higher relative importance scores than other features used in model training. (**D**) The model trained on 23 parameters achieved an F1 score of 0.67 and an accuracy of 0.84. *Note* High-density lipoprotein (HDL) cholesterol, low-density lipoprotein (LDL) cholesterol. RDW for red blood cell distribution width, RBC for red blood cell counts, MCV for mean corpuscular volume, ALT for alanine transaminase, MCHC for mean corpuscular hemoglobin.

**Figure 3:**
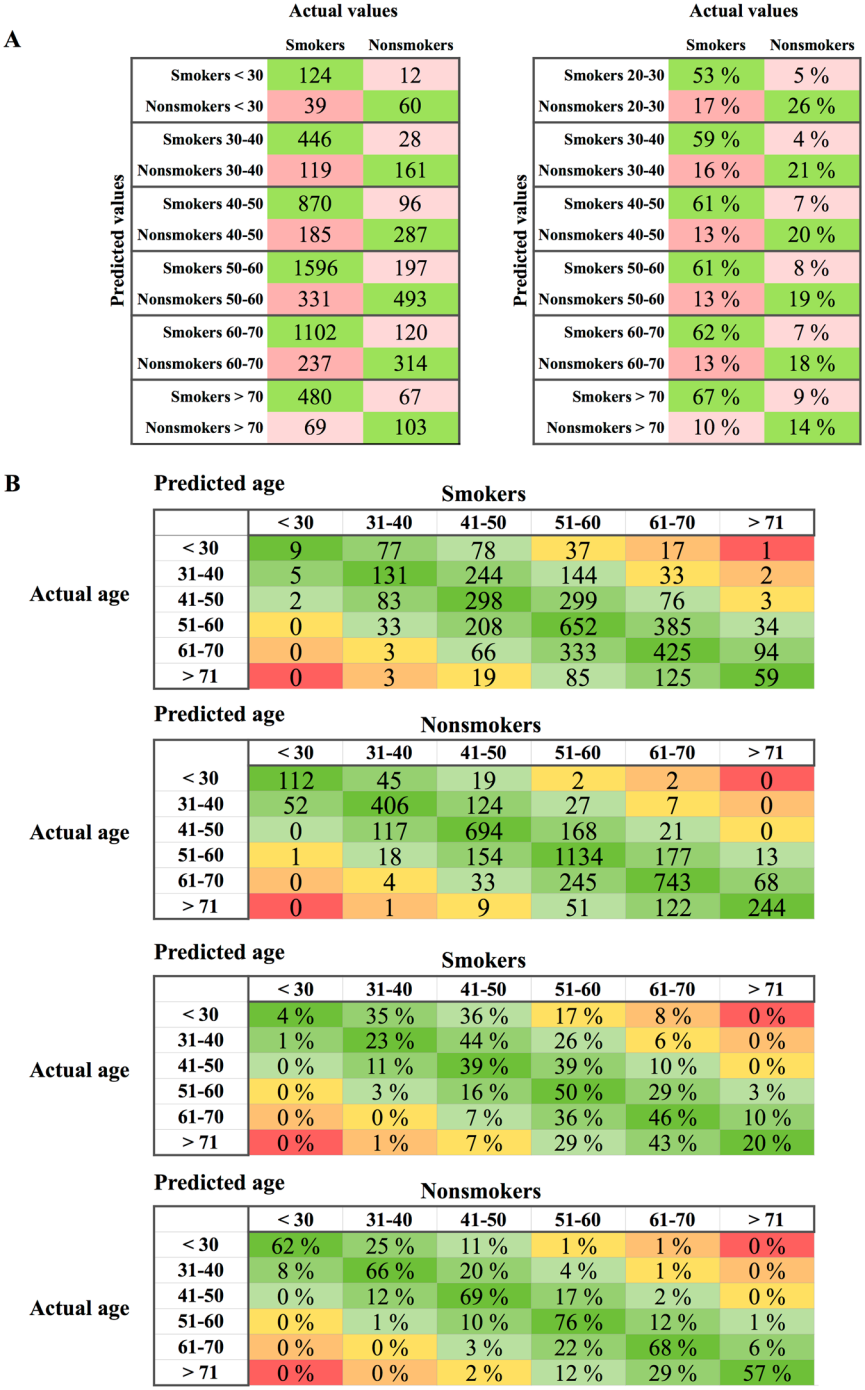
Confusion matrices. (**A**) Confusion matrices for the best-performing smoking status classifier, trained on 23 features, in number of samples (left) and percentage (right). Row values show predicted smoking status, and columns show actual smoking status. Most of the error smoking predictions occurred in individuals older than 55 years. (**B**) Confusion matrices for age prediction by age groups for the best model, trained on 24 parameters, in number of samples (left) and percentage (right). Row values show actual chronological age group, and columns show predicted age group. Smokers of age groups < 30 and 30–40 were mostly predicted to be older.

To investigate the effect of smoking on age prediction, we used neural networks trained on nonsmokers to calculate the age of the smokers and nonsmokers excluded from the training set. Model demonstrated R^2^ of 0.57 in predicting non-smokers and R^2^ of 0.55 in predicting smokers. We also calculated the log_2_ aging ratio (Formula 5) as proposed by Hannum *et al*.^14^. Compared with nonsmokers, smokers showed an accelerated rate of aging through to age 55 years regardless of sex (Figs 2B and 3, Supplementary Fig. 8). After age 55, these differences disappeared and perhaps even reversed themselves for the most elderly subjects (Figs 2B and 3, Supplementary Table 4). In the context of biological aging, this suggests that the contribution of tobacco smoking as an external factor of aging may eventually be masked by the intrinsically stochastic and physiologically deleterious nature of the aging process. Alternatively, the people most affected by smoking may have died at an earlier age and thus were be excluded from the old-age smoking group.

To further evaluate the importance of smoking status in age prediction we included smoking status as an input feature along with blood test values and trained the new set of DNNs on the three extended sets of input features. Smokers were included in the training set for this round. To robustly compare the performance of these models with models trained on nonsmokers, we used the same number of samples in the training sets. The best-performing deep neural network, which was trained on 24 blood test input features, performed better than the model trained on 23 input features (without smoking status) and achieved an *R*^2^ of 0.60 and an *MAE* of 5.42 years (Fig. 2A, Table 1). Deep neural networks trained on 21 and 19 blood test input features also exhibited higher age-prediction accuracy than the models trained on 20 and 18 blood test input features, respectively (Supplementary Figs S5A and S5B, Table 1). These results suggest that smoking status plays an important role in predicting age. However, this feature was not among the five most important features (Supplementary Figs S5C, S5D and 5E). To evaluate the dependence between age prediction as a target function and smoking status, we conducted a partial dependence analysis that confirmed predicted age increase with a smoking status of 1 (smokers) (Supplementary Figs 7–9). The same analysis of sex as an input feature showed that predicted age increases slightly with a sex of 1 (male) (Supplementary Fig. 9).

**Table 1:** Prediction accuracy of the three top-performing models after rounds of optimization.

	No. of features	*MAE* (years)	*r*	*ε-accuracy* (*ε* = 10 years)	*R* ^2^
Age predictor trained on 23 features	23	5.722	0.76	0.803	0.56
Age predictor trained on 20 features	20	5.777	0.75	0.801	0.5376
Age predictor trained on 18 features	18	5.898	0.75	0.802	0.55
Age predictor trained on 24 features	24	5.61	0.78	0.82	0.578
Age predictor trained on 21	21	5.401	0.77	0.815	0.58
Age predictor trained on 19 features	19	5.416	0.77	0.817	0.60
	**No. of features**	***Accuracy***	***Precision***	***Recall***	***F1***
Smoking status classifier trained on 23 features	23	0.829	0.754	0.606	0.673
Smoking status classifier trained on 20 features	20	0.822	0.726	0.61	0.664
Smoking status classifier trained on 18 features	18	0.82	0.708	0.603	0.638

### Deep-learned biochemistry clocks as biomarkers of lifestyle

To explore whether the smoking status of patients could be assessed using only patient sex and their blood test values we trained three DNNs on the same input feature sets used in the prior models to classify smokers and nonsmokers. The best-performing smoking status classifier, which was trained on 23 blood test input features, achieved an accuracy of 0.83 and an F1 score of 0.67, followed in descending order by the model trained on 20 blood test input features, and the model trained on 18 blood test input features (Fig. 2D, Supplementary Figs 6A,B, Table 1). High-density lipoprotein (HDL) cholesterol, hemoglobin, RDW, and mean corpuscular volume (MCV) were consistently the most important factors in determining a patient’s smoking status (Fig. 2C, Supplementary Fig. 4C,D).

Curiously, most of the false-positive and false-negative smoking status predictions occurred in individuals older than 55 years (Fig. 3A). This observation was consistent with the increased error rate that accompanied predictions of the ages of smokers and nonsmokers who were chronologically younger than 40 years. Furthermore, the majority of smoker samples for individuals younger than 30 years were predicted to be within the range of 31–40 years (35%) and 41–50 years (36%), whereas the ages of most of the nonsmokers (62%) were predicted correctly (Fig. 3B). The same trend was observed for the 31–40 age group, in which the ages of 43% of the smokers were predicted to be 41–50, and only 23.43% of nonsmokers were predicted to fall within the 31–40 age group. This trend was not observed in subjects older than 51 years and was therefore consistent with the observation made above.

### Cardiovascular disease risk and smoking status

**Figure 4:**
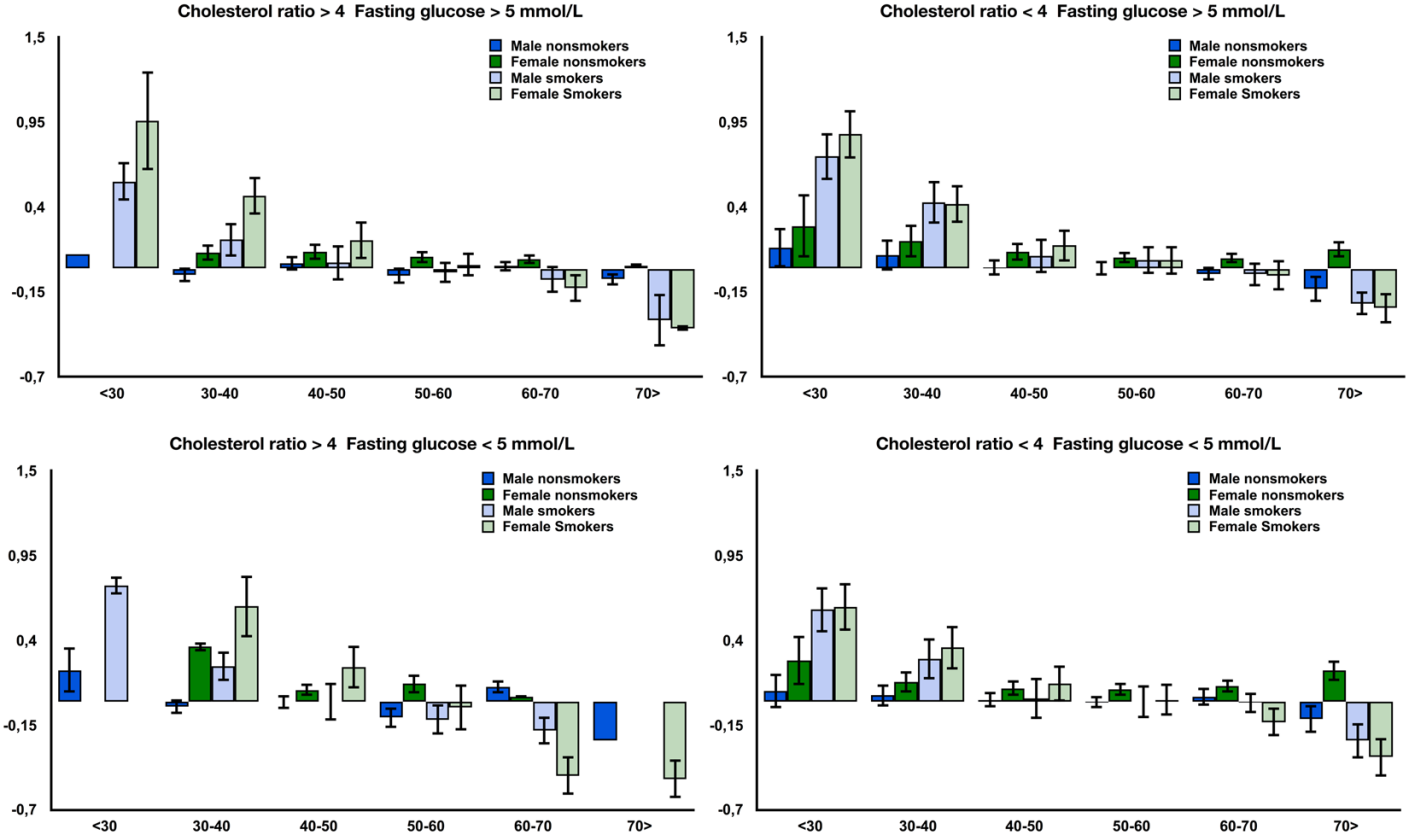
Log_2_ aging ratios for the four groups Cholesterol ratio > 4 and Fasting Glucose > 5 mmol/L, Cholesterol ratio > 4 and Fasting Glucose <= 5 mmol/L, Cholesterol ratio <= 4 and Fasting Glucose > 5 mmol/L, and Cholesterol ratio > 4 and Fasting Glucose > 5 mmol/L. Smokers of age groups < 30 and 31–40 are predicted older regardless their Cholesterol ratio and Fasting Glucose level. Log_2_ aging ratio of 1 means that sample is predicted two fold older than a chronological age and log_2_ aging ratio of −1 means sample is predicted half as old. Bars indicate standard deviation.

To assess the cardiovascular risk values, we examined the cholesterol ratio, which was calculated by dividing total cholesterol by HDL cholesterol (*cholesterol ratio* = *total cholesterol/HDL cholesterol)*. We classified the blood samples into four groups based on their cholesterol ratios and fasting glucose levels, using the following reference ranges: (1) cholesterol ratio > 4 and fasting glucose >5 mmol/L; (2) cholesterol ratio > 4 and fasting glucose ≤ 5 mmol/L; (3) cholesterol ratio 4 and fasting glucose > 5 mmol/L; and (4) cholesterol ratio ≤ 4 and fasting glucose > 5 mmol/L. As shown in Fig. 4, smokers had a higher log_2_ aging ratio than did nonsmokers regardless of their cholesterol ratio and fasting glucose levels. On average, female smokers were predicted to be twice as old as their chronological age as compared to non-smokers. Male smokers, on average, were predicted to be one and a half times as old as their actual chronological age compared to nonsmokers. However, females with cholesterol ratio > 4 and fasting glucose < 5 mmol/L tended to be predicted as being older. Interestingly, our results also suggest that smokers from the age groups 60–70 years and >70 years with a normal glucose level (<5 mmol) are predicted to be younger than their chronological age. This phenomenon is not observed in smokers with a high blood glucose level.

## Discussion

Our study, based exclusively on the analysis of routine blood test results, identifies complex nonlinear interactions between these test results, aging, and smoking status. Previous studies demonstrated that smoking exacerbates epigenetic aging^15,17^, but our study is the first to use blood test results to quantify this effect. Although our hematological aging clocks are slightly less accurate in chronological age prediction than DNA-methylation-based predictors^18,19^, our method they are less expensive and more practical requiring only standard blood tests.

Our study also demonstrated that young smokers (<40 years of age) have biological ages that are significantly higher than their chronological ages. Surprisingly, this effect disappears in the oldest subjects. At the same time, the study conducted by Levine and Crimmins (2016) showed similar results^16^. They showed that smokers from the 80 years old age group have no increase in mortality risk compared to smokers from other age groups. This could suggest that susceptible elderly smokers may have died off as a consequence of their smoking habits. An alternative hypothesis is that tobacco smoking may stimulate the activation of repair processes; his phenomenon has been proposed as a potential mechanism of tobacco-smoking protection from Parkinsons disease^20^.

Deep learning-based hematological aging clocks can serve as reasonably accurate predictors of age for relatively healthy individuals. These clocks can also serve as accurate tools for evaluating the effect of lifestyle factors (such as tobacco use) on biological aging. Furthermore, they can act as accurate classifiers of patient smoking status. Classifiers based on deep neural networks have the potential to support or even replace patient self-reporting and can thereby provide a better statistical assessment of the prevalence of tobacco smoking. The deep learning–based approach used in this study may be extended to analyze the combined effects of tobacco smoking and biochemically-defined diabetes mellitus and dyslipidemia as well as other potential morbidities. Similarly, DNNs could be used to predict health trajectories and outcomes or to evaluate the extent to which various other environmental exposures, dietary factors, and genetic risks affect health and aging.

## Materials and Methods

### Data overview

Following the Provincial REB approval by the Human Ethics Research Board of Alberta. Community Health Committee (HREBA.CHC-16-0066), the administrative dataset consisted of fully-anonymized records for 149,000 adult subjects. Informed consent was waived by the HREBA.CHC because the study was based on the fully-anonymized administrative dataset from the Analytics and Performance Reporting Branch of the Alberta Health Services. The study was performed is accordance with the HREBA.CHC guidelines and regulations outlined in the HREBA.CHC-16-0066 approved protocol. Each record included smoking status, sex, age, and up to 66 blood biochemistry and hematology markers. Of the 149,000 subjects, 33% (49,000) reported being smokers. Smokers and nonsmokers were matched for age distribution, sex, urban versus rural residence, and geographical latitude of residence. As per HREBA protocol, we did not have any information on either racial or ethnic origins, and analysis of any racial or ethnical effects was not permitted.

#### Training and test-set design

Blood biochemistry datasets were first preprocessed and normalized as previously described^8^. We treated the age prediction as a regression task. We split the data into the training and test sets at an 80/20 ratio. The deep neural network was built by adjusting its hyperparameters (e.g., number of layers, activation function, etc.) on the training set and subsequently measuring the performance of the trained neural network on the test set.

#### Feature reconstruction

To expand the feature space used to train our predictors, we applied regression and reconstructed missing values for part of the analyzed dataset. Between 30% and 60% of the dataset (depending on which of the three feature spaces we considered) was used to fit the linear regression of a given marker, and the results of this regression model were used to predict the missing values of each marker for the rest of the dataset. Marker values were reconstructed individually. Reconstruction of the missing values in this manner increased the size of each feature space from 14, 15, and 18 features to 18, 20, and 23 features, respectively.

#### DNN architectures

We used multilayer feed-forward back propagation neural networks as deep models (i.e., models with more than three layers). The Python 3.x (https://www.python.org) implementations of Keras (https://keras.io/) and Theano libraries (http://deeplearning.net/software/theano/) were used to build and train the neural networks. A grid search algorithm was used for multiple hyperparameters, optimizing for each feature space to achieve the greatest predictive accuracy. We minimized the *MAE* loss function using a back propagation algorithm. We used the S-shaped ReLU activation function^21^ in each layer, EVE^22^ or ADAM^23^ as optimizers of the cost function, and a dropout^24^ with 35% probability after each layer to ensure data regularization. We trained the networks with five fold cross-validation to compensate for overfitting and to achieve more robust performance metrics. The optimized architectures of each DNN are presented in Supp. Table 6.

To predict smoking status, we trained three classifiers on three different feature spaces. To do so, we again used simple feed-forward back propagation neural networks as deep models. Multiple hyperparameters were adjusted for each feature space to achieve the greatest predictive accuracy. We minimized binary cross-entropy loss function via the use of a back propagation algorithm. We used the S-shaped ReLU activation function in each layer, EVE or ADAM as optimizers of the cost function, and a dropout with 35% probability after each layer. We trained each network with fivefold cross-validation to compensate for overfitting and to achieve more robust performance metrics. The optimized architectures of each DNN are presented in Supplementary Table 6.

All experiments were conducted on a machine with Intel Xeon CPU E5-2660 with 256GB of RAM and NVIDIA Titan X (Pascal). Models were trained with early stopping with the average training time of 25 min.

#### Feature importance evaluation

For the FI evaluation, we used an RF FI ranking for the feature selection and permutation feature importance (PFI) for the final ranking. The Python scikit-learn library was used to train RF models^25^. The RF technique allows features to be ranked according to the decrease in accuracy averaged by each set of tree values (i.e., each tree predicts age according to one marker and assigns an importance coefficient to the marker; each prediction is summed, and each marker-associated importance factor is averaged to yield the final value). We trained 320 RF models on distinct feature spaces using 80 decision-tree estimators, with some hyperparameters adjusted and others set to default. For each feature, we adjusted the relative importance of the *MAE* score for the model (Formula 1):

1 $$FI=\sum _{i=1}^{N}\,\frac{{q}_{i}}{MA{E}_{i}}$$

where *q*_*i*_ is the mean decrease accuracy of the *i* model and *MAE*_*i*_ is the mean absolute error of the *i* model.

PFI is a wrapper method that we previously applied to determine the list of the most important blood test features for age prediction^12,13^. We applied the same technique for the age-prediction and smoking-status-prediction models discussed in the present study.

#### Statistical analysis

R library “WGCNA”^26^ was used for the calculation of biweight mid-correlation. The function “wilcox.test” from the “stat” R package (https://stat.ethz.ch/R-manual/R-devel/library/stats/html/wilcox.test.html) was used to perform a two-tailed Mann-Whitney nonparametric test of log_2_ aging ratio for smokers and nonsmokers. If *p*-values were less than 0.05, we would reject the null hypothesis.

#### Evaluation metrics

The following metrics were used to evaluate the predictive accuracy of the age-prediction and smoking-status-prediction models:

2 $${\rm{Pearson}}\,{\rm{correlation}}\,{\rm{coefficient}}:r=\frac{{\sum }_{i=1}^{N}\,({x}_{i}-\bar{x})({y}_{i}-\bar{y})}{\sqrt{{\sum }_{i=1}^{N}\,{({x}_{i}-\bar{x})}^{2}}\sqrt{{\sum }_{i=1}^{N}\,{({y}_{i}-\bar{y})}^{2}}},$$

where *x*_*i*_ is chronological age value and *x*′ is the mean of *x*, *y*_*i*_ is predicted age value and *y*′ is the mean of *y*, *N* is number of samples; *r* shows the strength of a linear association between predicted and actual age.

3 $${\rm{Coefficient}}\,{\rm{of}}\,{\rm{determination}}:{R}^{2}=1-\frac{{\sum }_{i=1}^{N}\,{({\hat{y}}_{i}-{y}_{i})}^{2}}{{\sum }_{i=1}^{N}\,{({y}_{i}-y^{\prime} )}^{2}},$$

where *y*_*i*_ is the real value, ${\hat{y}}_{i}\,$is the predicted value, and $\underline{y^{\prime} }$ is the mean of *y*. *R*^2^ shows the percentage of variance explained by the regression between predicted and actual age.

4 $${\rm{Mean}}\,{\rm{absolute}}\,{\rm{error}}:MAE=\frac{1}{N}\sum _{i=1}^{N}|{\hat{y}}_{i}-{y}_{i}|,$$

where ${\hat{y}}_{i}$ is a predicted age, *y*_*i*_ is an age value, and *N* is a number of samples. *MAE* demonstrates average disagreement between the chronological age and the predicted age.

5 $$\mathrm{Log}\,2\,{\rm{transformed}}\,{\rm{aging}}\,{\rm{ratio}}:lo{g}_{2}Aging\,ratio=lo{g}_{2}(\frac{{\hat{y}}_{i}}{{y}_{i}}),$$

where ${\hat{y}}_{i}$ is an age prediction of the model, *y*_*i*_ is an actual chronological age value, and *N* is a number of samples. Aging ratio is the ratio of predicted age to observed chronological age. A log_2_ aging ratio of 1 means the sample is predicted to be twofold older than a chronological age, and an log_2_ aging ratio of −1 means the sample is predicted to be half as old as a chronological age.

6 $$\varepsilon -accuracy=\frac{{\sum }_{i=1}^{N}\,{1}_{A}({\hat{y}}_{i})}{N},$$

where $A=[{y}_{i}-\varepsilon ;{y}_{i}+\varepsilon ]$, ${\hat{y}}_{i}$ is an age prediction of the model, and *y*_*i*_ is a true age value. For instance, if epsilon $(\varepsilon )\,$is 5 and the DNN model predicts an age of 55 but the real age is 50 or 60, then according to epsilon accuracy, such a sample would be considered correctly classified.

7 $${\rm{F}}1\,{\rm{score}}:F1=2\times \frac{precision\times recall}{precision+recall};$$

where precision and recall is calculated as:

8 $$precision=\frac{\sum tp}{\sum tp+\sum fp},$$

where is tp is true positive and fp is a false positive. Precision shows specificity of a model and equals to a fraction of correctly predicted smoker samples to the all samples predicted as smokers;

9 $$recall=\frac{\sum tp}{\sum tp+\sum fn},$$

where tp is a true positive, fn is a false negative. Recall shows the sensitivity of a model and equals to a fraction of correctly predicted smoker samples compared to all smoker samples.;

F1 score shows the weighted average of the *precision* and *recall*.

10 $${\rm{Accuracy}}:Accuracy=\frac{\sum tp+\sum tn}{\sum tp+\sum tn+\sum fp+\sum fn};$$

where tp is a true positive, tn is a true negative, fp is a false positive and fn is a false negative. Accuracy is a fraction of correctly predicted smoking status to the all values.

## Electronic supplementary material

Supplementary materials

## Data Availability

As per provisions of the strictly enforced Health Information Act of the Province of Alberta, Canada and decision of the Provincial Ethics Board, only aggregate result data may be presented in the manuscript, and the source fully anonymized administrative dataset containing individual blood test results constitutes private health information and will never be made public or deposited in any public database. Requests for access to data have to be directed to Dr. Kovalchuk and will be handled in accordance with the Provincial Health Information Act.
