# Supplementary material for: Blood Biochemistry Analysis to Detect Smoking Status and Quantify Accelerated Aging in Smokers
Source: Sci Rep. 2019 Jan 15;9:142. doi: 10.1038/s41598-018-35704-w (PMC6333803; doi:10.1038/s41598-018-35704-w)
Supplement: Supplementary file 1 — Supplementary materials [file 41598_2018_35704_MOESM1_ESM.pdf]

SUPPLEMENTARY INFORMATION

Blood Biochemistry Analysis to Detect Smoking Status and Quantify Accelerated Aging in Smokers

Polina Mamoshina,[1,2,3] Kirill Kochetov,[1,2,4] Franco Cortese,[5,6] Anna Kovalchuk,[2,7,8] Alexander Aliper,[1,2] Evgeny Putin,[1,2,4] Morten Scheibye-Knudsen[9], Charles R. Cantor[10],Neil M. Skjodt [2, 7], Olga Kovalchuk [2,7*,#], Alex Zhavoronkov[1,2,5, 11*,#]

Affiliations

1. Pharmaceutical Artificial Intelligence Department, Insilico Medicine, Inc., Emerging Technology Centers, Johns Hopkins University, Baltimore, Maryland 21218, USA
2. Canada Cancer and Aging Research Laboratories, Ltd, Lethbridge, Alberta T1K7X8, Canada
3. Computer Science Department, University of Oxford, Oxford, United Kingdom
4. Computer Technologies Lab, ITMO University, St. Petersburg 197101, Russia
5. Biogerontology Research Foundation, Research Department, Oxford, United Kingdom
6. Canadian Longevity Alliance, Ontario, Canada
7. University of Lethbridge, Lethbridge, Alberta T1K3M4, Canada
8. Leaders in Medicine Program, Cumming School of Medicine, University of Calgary, Alberta T2N 4N1, Canada
9. Center for Healthy Aging, Department of Cellular and Molecular Medicine, University of Copenhagen, Denmark
10. Boston University, Department of Biomedical Engineering, Boston, Massachusetts 02215, USA.
11. Buck Institute for Research on Aging, 8001 Redwood Boulevard, Novato, CA 94945, USA

*Correspondence should be addressed to: Olga Kovalchuk (olga.kovalchuk@ccarl.ca or olga.kovalchuk@uleth.ca) and Alex Zhavoronkov (alex@insilicomedicine.com)
# -These authors contributed equally to the research

Supplementary Methods:

1. Data overview and preprocessing

Following Provincial REB approval (HREBA.CHC-16-0066) fully anonymized administrative dataset containing fully anonymized records for 149000 adult subjects was obtained. Each record included smoking status, gender, age, and up to 66 blood biochemistry and haematology markers. Out of the 66 markers, 36 overlapped with the 41 features that our previous Aging.AI 1.0 system[1] has been trained upon. Eight of these markers contributed most to the predictive accuracy of our model (Supplementary Table 1). Thirty three percent (49000) (33%) of 149000 subjects smoked. Smokers and nonsmokers were matched for age distribution, gender, urban versus rural residence, and geographical latitude of residence (Supplementary Fig. 1). Age distribution tended to normal, with the majority of samples and the majority of smokers respectively within the 51-60 year-old age group.

For consistency, relevance and accuracy, and in order to obtain a sufficient sample size we first analysed the number of available samples measured at the same time, i.e. with the same test date, with the aim of constructing feature spaces to train our DNNs consisting exclusively of blood-based biomarkers measured on the same day.

We then applied arbitrage feature selection on different subsets with different numbers of measured markers to optimize the feature spaces used to train age prediction models . We trained 320 Random Forest (RF) models on distinct feature spaces extracting feature importance values (FIs) from each model. These FIs were combined considering model score (Supplementary Fig. 2). Predictor accuracy depends both on the sample size and its training feature space size. To increase the training feature space we applied regression to reconstruct missing values. This manner increased feature spaces sizes from 14, 15, and 18 features to 18, 20, and 23 respectively.

Glycated hemoglobin (Hemoglobin A1c) best predicted age followed by Urea, Fasting Glucose and Ferritin. Glycated hemoglobin is a well-known measure of long-term plasma glucose concentration and one of the most common measures of glucose control in diabetic patients [2, 3]. The concentration of glycated hemoglobin has also been shown to increase with age,[4] as has the concentration of other glycated plasma proteins.[5] Albumin was less predictive despite being most predictive in previous models derived with the Aging.AI system.[1] This discrepancy may be the result from different training set distributions used by the two predictors; the original population of Aging.AI has having a wider distribution and a larger dispersion of albumin.

The most predictive markers selected by FI arbitage ranking had only weak biocorrelation with age on their own (Supplementary Fig. 3 and Supplementary Table 2).

2. Deep Learned biochemistry clocks.

We excluded smokers from the initial training set. Using feature importance ranking as received by the set of RFs, we trained DNNs were trained on three different feature spaces using the maximum available number of samples containing the importance ranked features chosen via arbitrage selection for inclusion in each of the three feature spaces (Supplementary Table 3).

All three models highly correlated with chronological age. The three best performing DNNs were trained first on 23 parameters, with an *MAE* of 5.72 years (R^2=0.56), second on 20 parameters, with an *MAE* of 5.78(R^2 =0.578), and third on 18 parameters with an *MAE* of 5.90 years (R^2=0.55) (Fig. 1 A, Supplementary Fig. 4 A, B and Supplementary).

Fasting glucose was found to be the most important feature for all three models followed by gender (Fig. 1 C and Supplementary Fig. 4 C, D). Fasting glucose is among the most important markers used by our previously-reported Aging.AI 1.0 system[1].

3. Deep Learned biochemistry clocks as lifestyle biomarkers

Haemoglobin, RBC, WBC, HDL, and total cholesterol have been associated with smoking [6]. We aimed to quantify the biological age differences between smokers and non-smokers, and to demonstrate a blood biochemistry classifier of smoking status.

Using the validated age-predictors described above we then included smoking status as a feature and re-trained the DNNs on an extended feature space to predict the chronological age of the smokers excluded from the initial training set. Introducing smoking status improved predictive accuracy of all three models (Fig. 2 A, Supplementary Fig. 5 A, B and Table 1).

For the all three models fasting glucose was the most predictive feature(Supplementary Fig. 5 C, D, E).

To predict smoking status, we trained classifiers on the three features spaces initially selected for a regression task. The best performing classifier achieves 0.83 predictive accuracy and an *F1* score of 0.67, followed by the model trained on 20 features and the model trained on 18 features (Fig. 2 D, Supplementary Fig. 6 A, B and Table 1). HDL Cholesterol, Hemoglobin, RDW, and MCV were consistently most predictive all three models (Fig. 2 C and Supplementary Fig. 6 C, D).

Most false-positive smoking predictions were made for patients older than 50 years of age (Fig. 3). This is consistent with the difference in ageing rates between smokers and non-smokers our study, which plateaued after 55 years of age.

To evaluate biological age differences between smokers and non-smokers, we used the best performing DNNs trained on 18, 20 and 23 features, respectively, to predict the age of patients that were initially excluded from the training set. The smokers demonstrated a higher aging ratio and their predicted age was consistently and significantly higher than non-smokers, regardless of gender until chronologic age of 55 years.(Fig. 2 B and Supplementary Fig. 8, Table 1). In the context of biological aging, this finding suggests that contribution of tobacco smoking as an external factor of aging might become masked by the intrinsically

-

-related comorbidity.

Supplementary References:

1. Putin, E. *et al.* Deep biomarkers of human aging: Application of deep neural networks to biomarker development. *Aging* **8,** 1021–1033 (2016).
2. Beltran del Rio, M. *et al.* Glycated Hemoglobin, Plasma Glucose, and Erythrocyte Aging. *J. Diabetes Sci. Technol.* **10,** 1303–1307 (2016).
3. Use of glycated haemoglobin (HbA1c) in the diagnosis of diabetes mellitus. *Diabetes Res. Clin. Pract.* **93,** 299–309 (2011).
4. Nakashima, K., Nishizaki, O. & Andoh, Y. Acceleration of hemoglobin glycation with aging. *Clin. Chim. Acta* **215,** 111–118 (1993).
5. Krone, C. A. & Ely, J. T. A. Ascorbic acid, glycation, glycohemoglobin and aging. *Med. Hypotheses* **62,** 275–279 (2004).
6. Aula, F. A. & Qadir, F. A. Effects of Cigarette Smoking on Some Immunological and Hematological Parameters in Male Smokers in Erbil City. *Jordan Journal of Biological Sciences* 159–166 (2013).

Supplementary figure legends:

Supplementary Figure 1. Number of samples by age and gender. The distribution tends to normal. Markers were normalized as described in Putin et. al,[1] and outliers with critical values (i.e. values indicative of laboratory errors) were excluded with the aim of reducing bias in the training set and increasing the biological accuracy of the predictors.

Supplementary Figure 2. Hemoglobin A1c is the most important marker, followed by Urea, Fasting Glucose and Ferritin.

Supplementary Figure 3. Ranked aging predictive blood markers stratified by smoking and gender. Fasting glucose demonstrates stronger correlation with age (*bicor=0. 27*) than other parameters. Total Cholesterol (*bicor=-0.07*) and AST (*bicor= 0.016*) demonstrate no correlation with age. Hemoglobin and RBC levels clearly separate female and male samples for the patients younger than 40 years old.

Supplementary Figure 4. Results of the best performing models trained on two feature sets. (A) The model trained on 18 features archives 5.90 years *MAE*(R^2 =0.55), (B) The model trained on 20 features archives 5.78 years *MAE* (R^2 = 0.536) (C) Feature importance plot for 18 features model; Hemoglobin, HDL Cholesterol and RDW are the three most predictive markers. (D) Feature importance plot for the model trained on 20 features.

Supplementary Figure 5. Results of the best performing models trained on two feature sets extended with smoking status. (A) The model trained on 19 features archives 5.61 years *MAE* and R^2 of 0.578. (B) The model trained on 21 features archives 5.401 yeas *MAE* and R^2 of 0.58. (C) Feature importance plot for the model trained on 19 features. (D) Feature importance plot for the model trained on 21 features. (E) Feature importance plot for the model trained on 24 features.

Supplementary Figure 6. Results of the best performing classifiers trained from two models.

(A) The model trained on 18 features archives an *F1* of 0.638. (B) The model trained on 20 features archives an *F1* of 0.664. (C) Feature importance plot of the model trained on 18 features. (D) Feature importance plot of the model trained on 20 features.

Supplementary Figure 7. The log2 aging ratio for smokers and non-smokers by age and gender groups for two best performing models. Smokers are predicted significantly older before 55 years old.

Supplementary Figure 8. Partial dependence plots between the age prediction and sex and smoking status input features. Smoke of 0 indicates non-smokers, smoke of 1 indicates smokers. Sex of 1 indicated males, sex of 0 indicated females.

Supplementary Figure 9. Distributions of predicted and actual ages of smokers and non-smokers for each sex and age groups.

Supplementary table legends:

Supplementary Table 1. Names and Codes of provided blood biochemistry and cell count markers.

Supplementary Table 2. The most important markers statistics, reference values and number of samples grouped by age group, smoking status and sex.

Supplementary Table 3. The number of features, list of features, sample size before exclusion of outliers and sample size after exclusion of outliers for the three best-performing models.

Supplementary Table 4. Aging ratio statistic for samples grouped by age group, sex and smoking status with W statistic values and p-values for smokers and nonsmokers and number of samples.

Supplementary Table 5. The optimized architectures of each DNN.

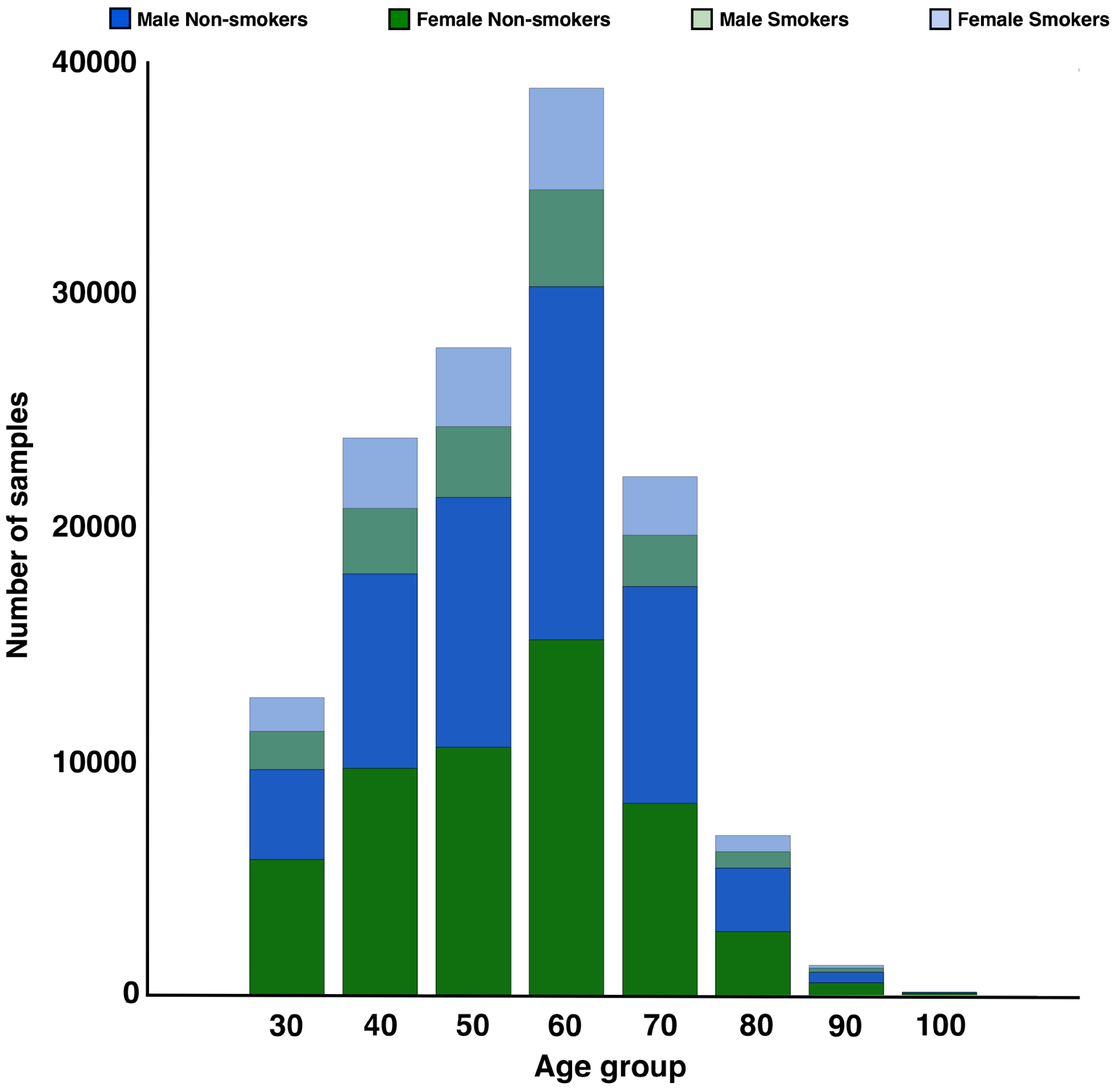

Supplementary Figure 1

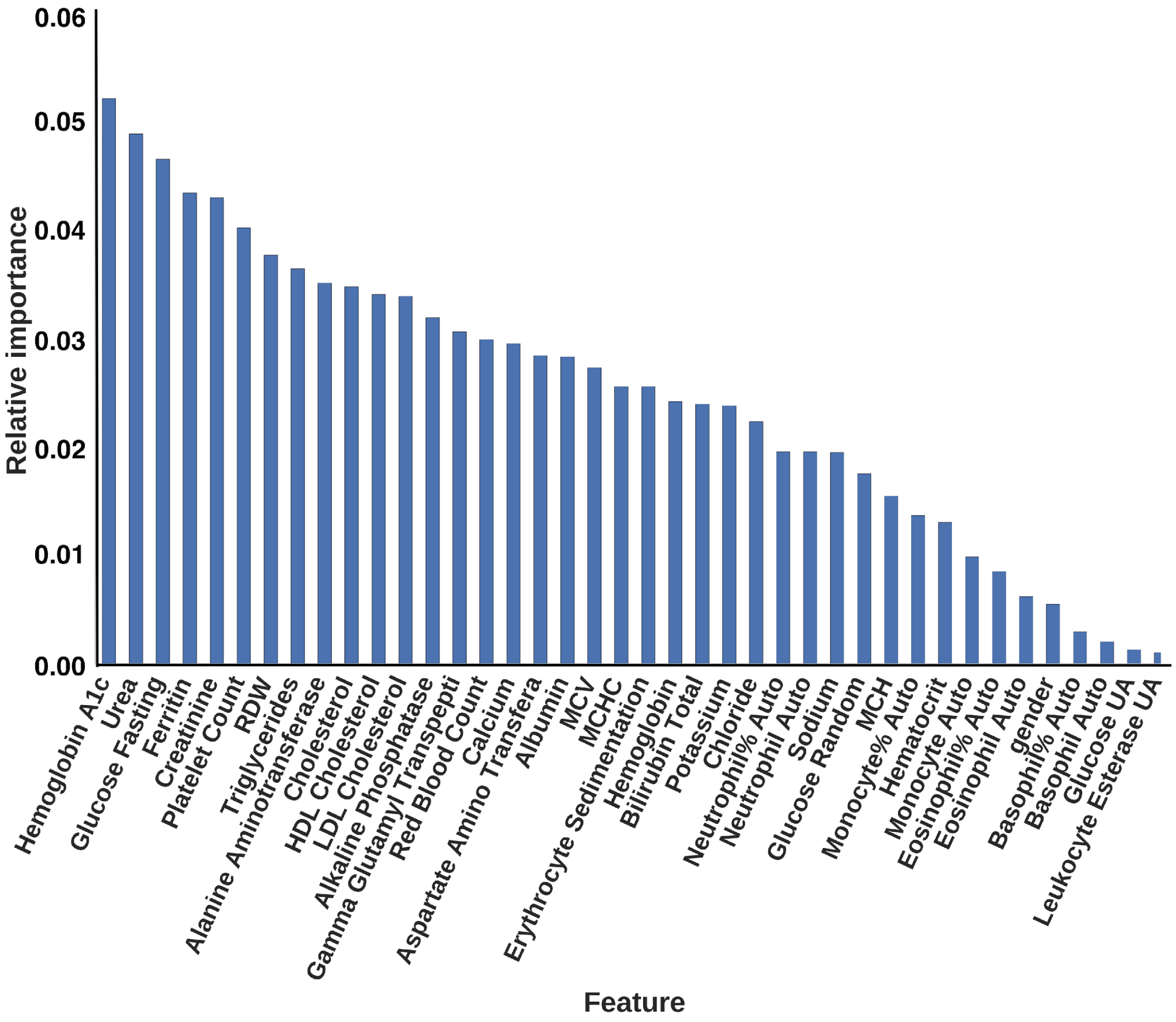

Supplementary Figure 2

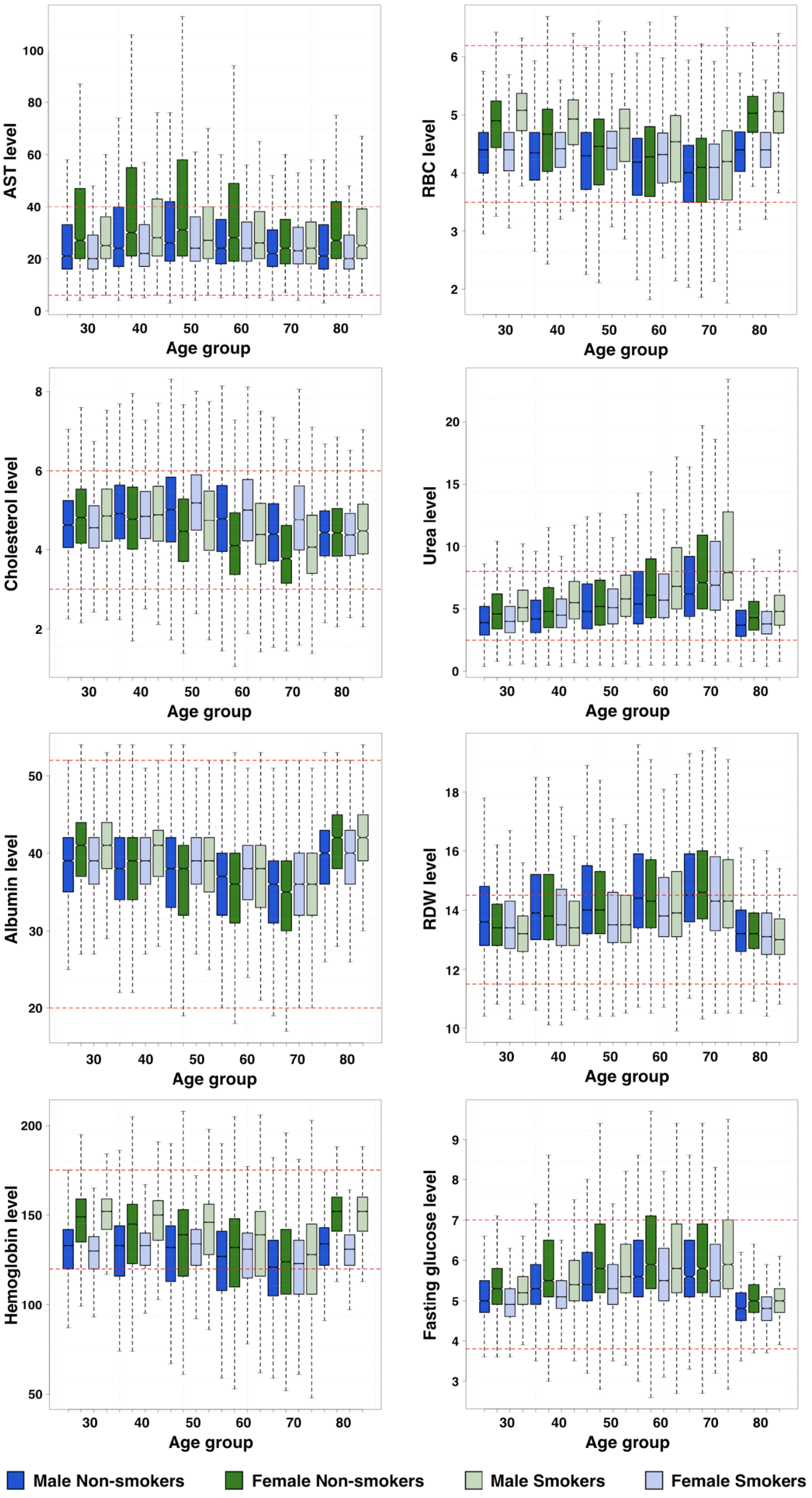

Supplementary Figure 3

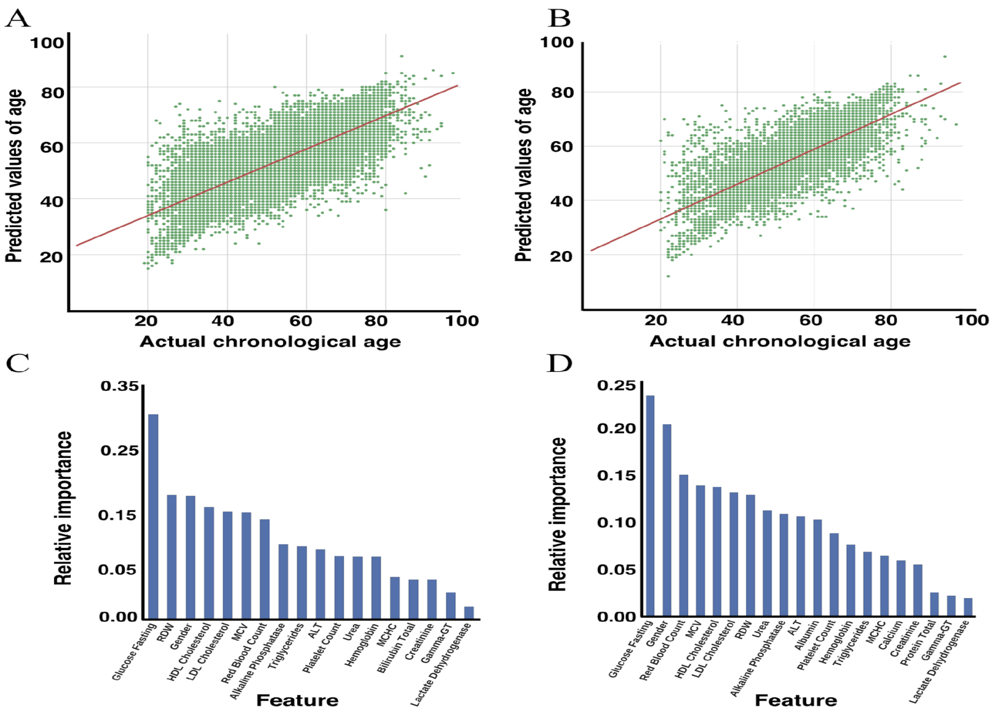

Supplementary Figure 4

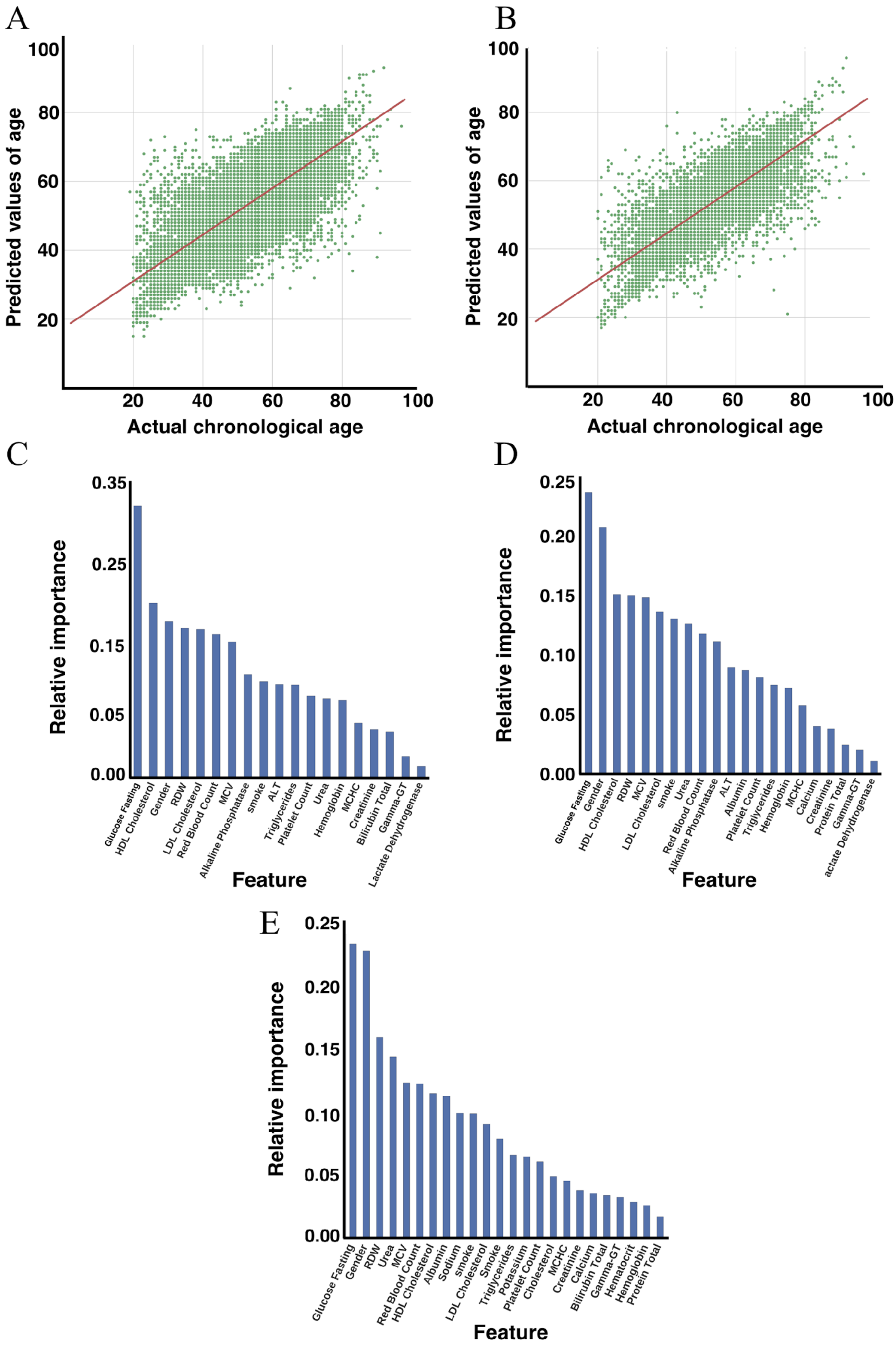

Supplementary Figure 5

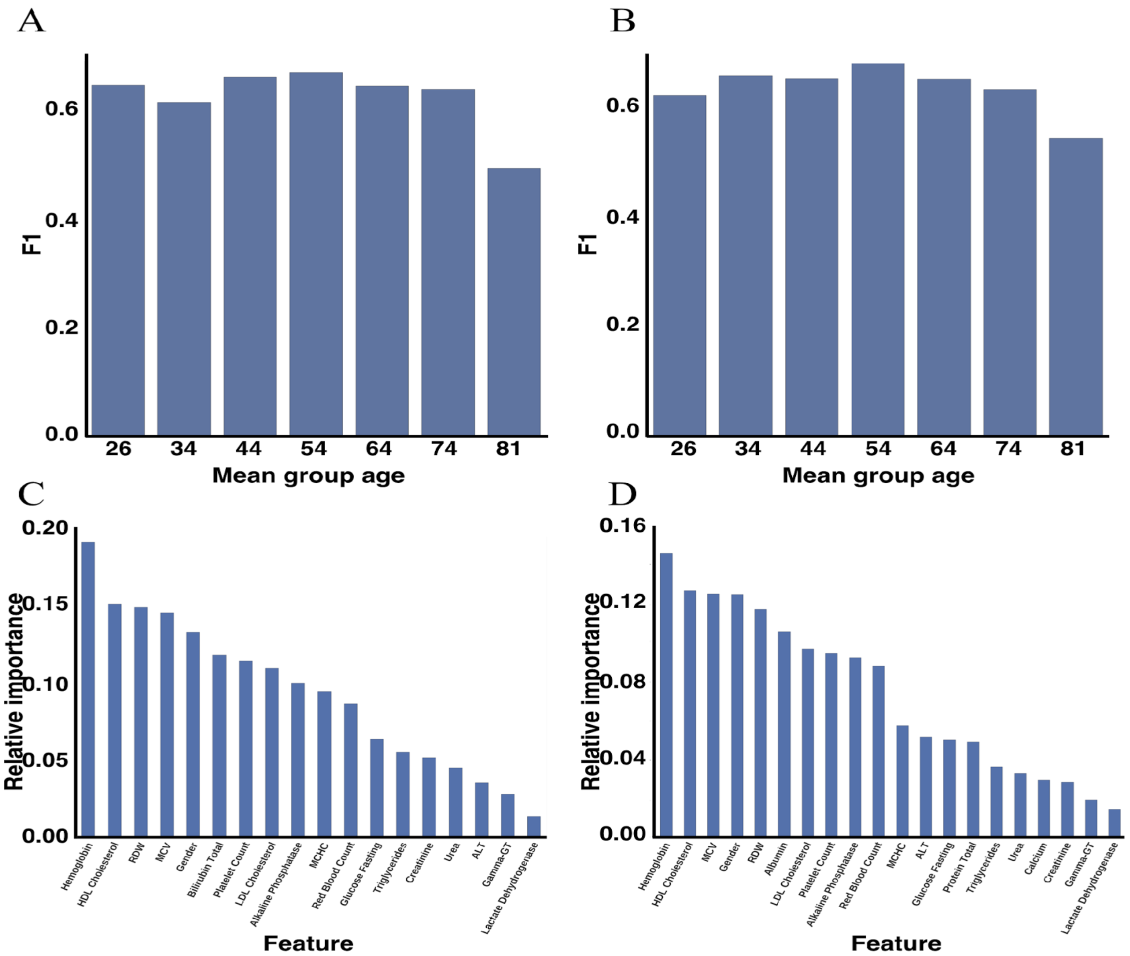

Supplementary Figure 6

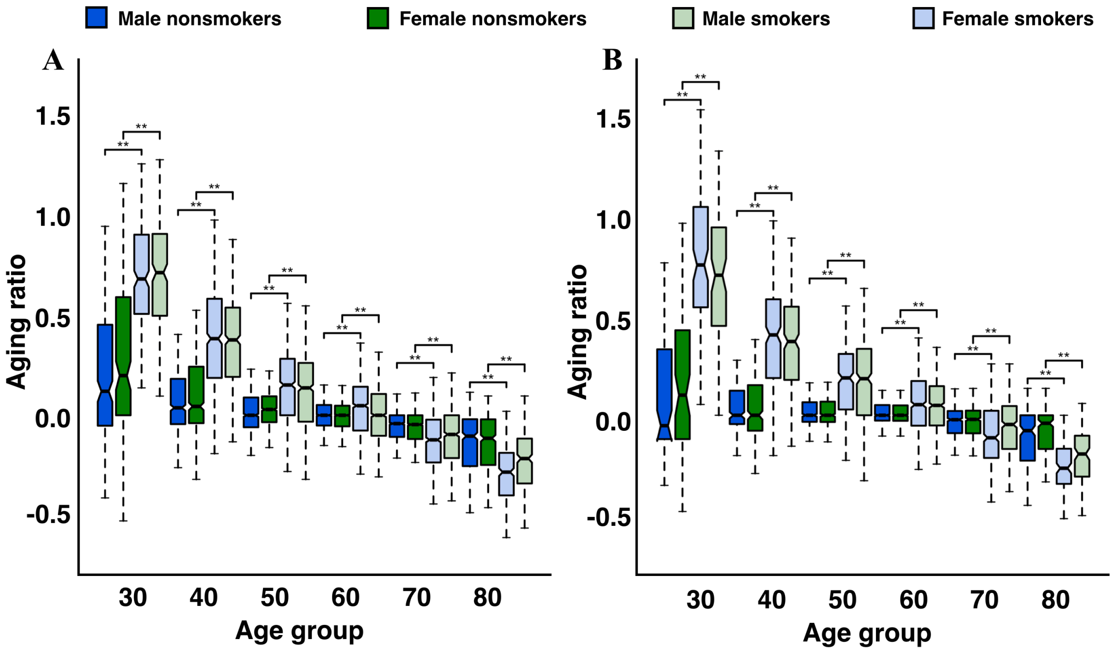

Supplementary Figure 7

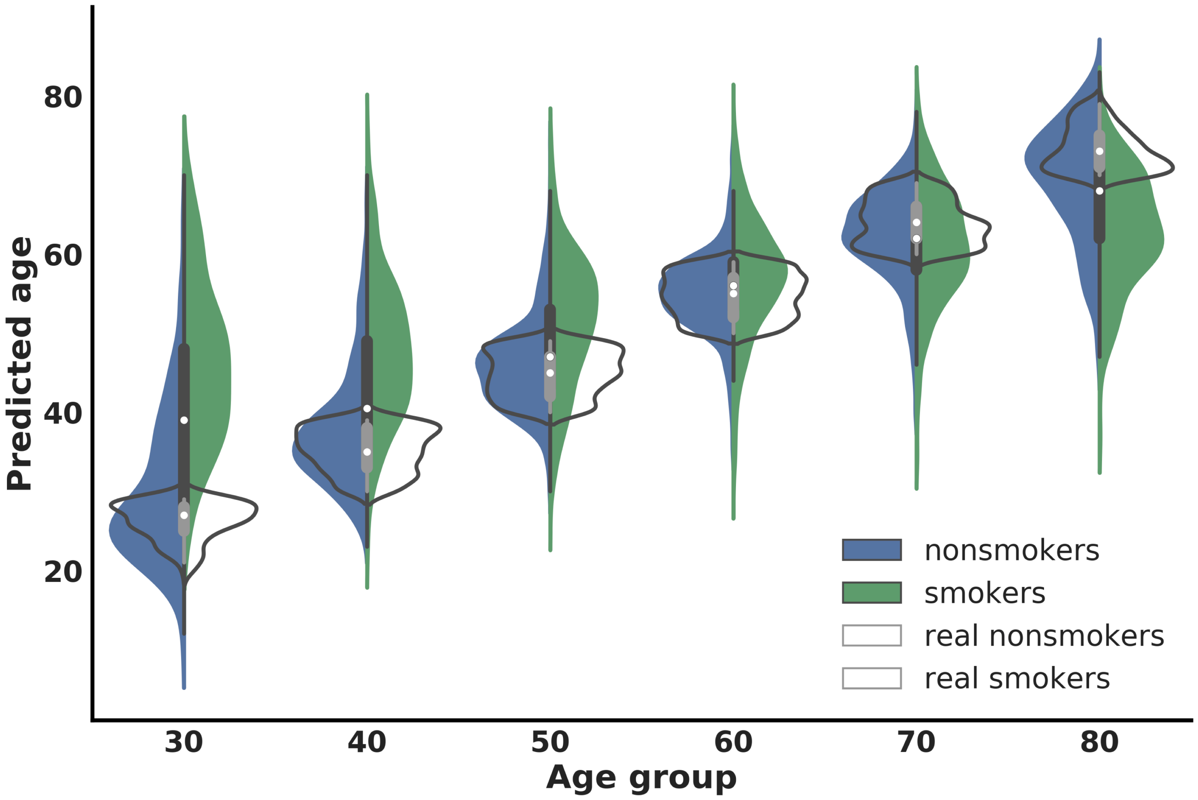

Supplementary Figure 8

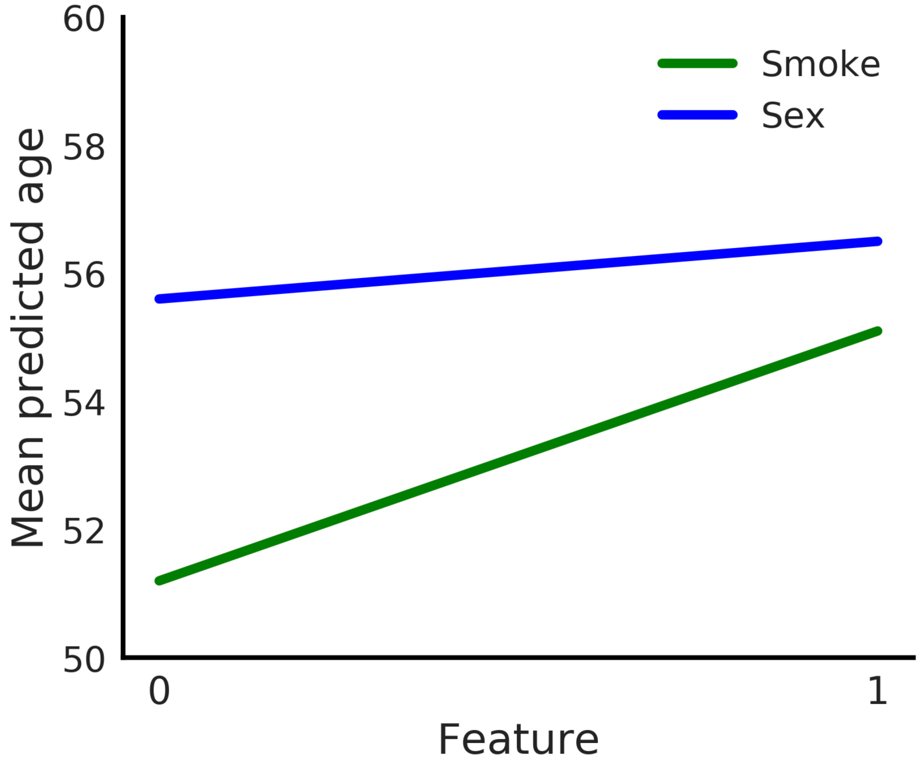

Supplementary Figure 9

Table S1. Names and Codes of provided blood biochemistry and cell count markers.

Data - Test Code	Test Name
ALB	Albumin
GLUF<br>GLUUA<br>316558.00	Glucose Fasting<br>Glucose UA<br>Glucose Random
ALP	Alkaline Phosphatase
UREA	Urea
RBC	Red Blood Count
CHOL	Cholesterol
RDW<br>RDWCV<br>4462477.00	RDW<br>RDW CV<br>RDW HE
HCT	Hematocrit
AMY<br>315952.00	Amylase
ESR	Erythrocyte Sedimentation
BILT	Bilirubin Total
GGT	Gamma Glutamyl Transpeptid
CREA<br>CREUR<br>CREU	Creatinine
LD	Lactate Dehydrogenase
TP	Protein Total
GLOBA1	GLOBULIN ALPHA 1
GLOBB	GLOBULIN BETA
SFIGG<br>IGG	IgG, CSF
SFIGG<br>IGG	IgG, CSF<br>IMMUNOGLOBULIN G
TRIG	Triglycerides
CL	Chloride
HDL	HDL Cholesterol
LDL	LDL Cholesterol
CA	Calcium
K	Potassium
NA	Sodium
FERR	Ferritin
HGB<br>HBA1C	Hemoglobin<br>Hemoglobin A1c
MCH<br>MCH2	Mean Corpuscular Hemoglobin,<br>MCH
MCHC<br>MCH2<br>316932.00<br>4462476.00	MeanCorpuscular HGB Conc<br>MCHC<br>MCHC<br>MCHC HE
MCV	Mean Corpuscular Volume

PLT	Platelet Count
LEUKUA	Leukocyte Esterase UA
ALT	Alanine Aminotransferase
AST	Aspartate Amino Transferase
BA#<br>316016.00<br>4382075.00	Basophil<br>Basophil% Auto<br>Basophil Auto
EO#<br>4382074.00<br>4382077.00	Eosinophil<br>Eosinophil Auto<br>Eosinophil% Auto
MO#<br>316968.00<br>4382073.00	Monocyte<br>Monocyte Auto<br>Monocyte% Auto
NE#<br>4382076.00<br>4382071.00<br>4382260.00	Neutrophil<br>Neutrophil Auto<br>Neutrophil% Auto<br>Hypersegmented Neutrophil

Table S2. The most important markers statistics, reference values and number of samples grouped by age group, smoking status, and sex.

Age group	Group	Number of sample	Lower whisker	Low reference value	Median	High reference value	Upper whisker
**AST (bicor = 0.01)**							
	Male non-smokers	4413	16		33		58
	Female non-smokers	3834	20		47		87
	Male smokers	4973	16		29		48
<30	Female smokers	3257	20		36		60
	Male non-smokers	5641	17		40		74
	Female non-smokers	7265	21		55		106
	Male smokers	6687	17		33		57
30-40	Female smokers	6563	21		43		76
	Male non-smokers	9588	19		42		76
	Female non-smokers	11703	21		58		113
	Male smokers	13016	19	6	36	40	61
40-50	Female smokers	12751	20		40		70
	Male non-smokers	5446	18		35		60
	Female non-smokers	6978	19		49		94
	Male smokers	8035	19		34		56
50-60	Female smokers	9576	20		38		65
	Male non-smokers	1968	17		31		52
	Female non-smokers	2066	18		35		60
	Male smokers	3870	18		32		53
60-70	Female smokers	4173	18		34		58
	Male non-smokers	2385	16		33		58
70>	Female non-	1978	20		42		75

	smokers						
	Male smokers	2860	16		29		48
	Female smokers	1425	20		39		67

RBC (bicor = -0.19)

	Male non-smokers	32262	2,95		4,4		5,75
	Female non-smokers	24573	3,25		4,9		6,43
	Male smokers	50746	3,05		4,4		5,69
<30	Female smokers	24438	3,78		5,08		6,33
	Male non-smokers	38041	2,65		4,35		5,93
	Female non-smokers	41119	2,43		4,67		6,7
	Male smokers	55994	3,2		4,42		5,6
30-40	Female smokers	45057	3,34		4,93		6,41
	Male non-smokers	62484	2,25		4,3		6,17
	Female non-smokers	73002	2,11		4,46		6,62
	Male smokers	93099	3,07		4,43		5,71
40-50	Female smokers	89191	2,86	3,5	4,77	6,2	6,44
	Male non-smokers	42686	2,16		4,19		6,06
	Female non-smokers	52877	1,82		4,28		6,6
	Male smokers	65594	2,54		4,32		5,98
50-60	Female smokers	77426	2,14		4,54		6,7
	Male non-smokers	24150	2,03		4,01		5,94
	Female non-smokers	23130	1,86		4,1		6,23
	Male smokers	41399	2,13		4,1		5,92
60-70	Female smokers	41847	1,76		4,2		6,51
	Male non-smokers	18517	3,02		4,4		5,72
	Female non-smokers	12732	3,77		5,03		6,25
70>	Male smokers	28711	3,2		4,4		5,6

	Female smokers	10031	3,66		5,06		6,41

Cholesterol (bicor = -0.07)

	Male non-smokers	5164	2,25		4,62		7,05
	Female non-smokers	5872	2,15		4,82		7,6
	Male smokers	11524	2,42		4,55		6,74
<30	Female smokers	10027	2,22		4,86		7,53
	Male non-smokers	8816	2,23		4,92		7,69
	Female non-smokers	10584	1,69		4,78		7,95
	Male smokers	22148	2,5		4,85		7,28
30-40	Female smokers	21096	2,11		4,89		7,71
	Male non-smokers	14727	1,72		5,02		8,31
	Female non-smokers	16601	1,38		4,46		7,67
	Male smokers	39191	2,38		5,19		8,01
40-50	Female smokers	38654	1,72	3	4,74	6	7,75
	Male non-smokers	8664	1,44		4,79		8,14
	Female non-smokers	10847	1,05		4,1		7,28
	Male smokers	24388	1,88		5,01		8,12
50-60	Female smokers	28208	1,42		4,38		7,5
	Male non-smokers	3135	1,53		4,39		7,35
	Female non-smokers	3297	1,44		3,77		6,79
	Male smokers	9254	1,59		4,76		8,06
60-70	Female smokers	9669	1,38		4,06		7,1
	Male non-smokers	2130	2,15		4,43		6,68
	Female non-smokers	2003	2,05		4,42		6,85
	Male smokers	4343	2,28		4,37		6,52
70>	Female smokers	2646	2,05		4,47		7,04

**Urea (bicor = 0.29)**							
	Male non-smokers	12821	0,4		3,9		8,6
	Female non-smokers	11293	0,8		4,6		10,4
	Male smokers	11289	0,5		4		8,3
<30	Female smokers	8376	0,6		5,1		10,2
	Male non-smokers	15109	0,4		4,2		9,6
	Female non-smokers	19592	0,5		4,8		11,5
	Male smokers	15244	0,4		4,5		9,2
30-40	Female smokers	16077	0,7		5,5		11,7
	Male non-smokers	26633	0,4		4,8		12,4
	Female non-smokers	34835	0,5		5,2		12,7
	Male smokers	27082	0,4		5,1		10,7
40-50	Female smokers	31712	0,6	2,5	5,8	8	12,6
	Male non-smokers	19276	0,4		5,4		14,3
	Female non-smokers	26421	0,5		6,1		16
	Male smokers	21137	0,5		5,7		13
50-60	Female smokers	30703	0,5		6,8		17,2
	Male non-smokers	11265	0,5		6,2		16,4
	Female non-smokers	11571	0,8		7,1		19,7
	Male smokers	16330	0,5		6,9		18,6
60-70	Female smokers	18035	0,8		7,9		23,4
	Male non-smokers	6555	0,4		3,7		8
	Female non-smokers	5748	0,8		4,3		9
	Male smokers	6328	0,4		3,8		7,5
70>	Female smokers	3750	0,8		4,8		9,7
**Albumin (bicor = -0.20)**							

	Male non-smokers	5599	25	20	39	50	52
	Female non-smokers	5043	27		41		54
	Male smokers	7472	27		39		51
<30	Female smokers	5508	29		41		53
	Male non-smokers	8221	22		38		54
	Female non-smokers	9448	22		39		54
	Male smokers	12494	27		39		51
30-40	Female smokers	10682	28		41		52
	Male non-smokers	14975	20		38		54
	Female non-smokers	17020	19		38		54
	Male smokers	23719	27		39		51
40-50	Female smokers	21178	25		39		52
	Male non-smokers	9668	20		37		52
	Female non-smokers	11267	18		36		53
	Male smokers	16271	24		38		51
50-60	Female smokers	18721	21		38		53
	Male non-smokers	4365	19		36		51
	Female non-smokers	4133	17		35		52
	Male smokers	9563	20		36		52
60-70	Female smokers	9437	20		36		51
	Male non-smokers	2565	26		40		53
	Female non-smokers	2108	28		42		53
	Male smokers	3553	26		40		52
70>	Female smokers	1948	30		42		54

Glucose Fasting (bicor=0.27)

	Male non-smokers	4278	3,6	3,8	5	7	6,6
<30	Female non-	4634	3,6		5,3		7,1

	smokers						
	Male smokers	9796	3,6		4,9		6,3
	Female smokers	7835	3,9		5,2		6,6
	Male non-smokers	7030	3,5		5,3		7,4
	Female non-smokers	8111	3		5,5		8,6
	Male smokers	18123	3,8		5,1		6,5
30-40	Female smokers	16521	3,5		5,4		7,5
	Male non-smokers	11820	3,2		5,4		8
	Female non-smokers	12978	2,8		5,8		9,4
	Male smokers	31642	3,5		5,3		7,4
40-50	Female smokers	30732	3,4		5,6		8,2
	Male non-smokers	6915	3		5,6		8,6
	Female non-smokers	8528	2,6		5,9		9,7
	Male smokers	19671	3,1		5,5		8,2
50-60	Female smokers	22946	2,7		5,8		9,4
	Male non-smokers	2465	3,3		5,6		8,6
	Female non-smokers	2610	2,7		5,8		9,4
	Male smokers	7606	3,2		5,5		8,3
60-70	Female smokers	7749	2,8		5,9		9,5
	Male non-smokers	1845	3,5		4,8		6,2
	Female non-smokers	1620	3,7		5		6,4
	Male smokers	3832	3,7		4,8		5,9
70>	Female smokers	2116	3,9		5		6,1
**RDW (bicor =0.22)**							
	Male non-smokers	23246	10,4		13,6		17,8
	Female non-smokers	16973	10,8	11,5	13,4	14,5	16,2
<30	Male smokers	29322	10,3		13,4		16,7

	Female smokers	14283	10,8		13,2		15,6
	Male non-smokers	26817	10,6		13,9		18,5
	Female non-smokers	28245	10,1		13,8		18,5
	Male smokers	33012	10,1		13,5		17,5
30-40	Female smokers	26551	10,6		13,4		16,5
	Male non-smokers	44504	10,3		14		18,9
	Female non-smokers	49444	10,4		14		18,4
	Male smokers	56222	10,4		13,5		17,1
40-50	Female smokers	53643	10,5		13,5		16,9
	Male non-smokers	29519	10,7		14,4		19,6
	Female non-smokers	34787	10,5		14,3		19,1
	Male smokers	40078	10,7		13,8		18,1
50-60	Female smokers	46760	9,9		13,9		18,6
	Male non-smokers	15568	11		14,5		19,3
	Female non-smokers	15303	10,3		14,6		19,4
	Male smokers	26084	10,5		14,3		19,5
60-70	Female smokers	26338	10,5		14,3		19,1
	Male non-smokers	13897	10,5		13,2		16,1
	Female non-smokers	9549	10,9		13,2		15,7
	Male smokers	18142	10,4		13,1		16
70>	Female smokers	6309	10,8		13		15,4

Hemoglobin (bicor= -0.15)

	Male non-smokers	32609	87		133		175
	Female non-smokers	24773	99		149		195
	Male smokers	51794	93	120	130	175	165
<30	Female smokers	24606	117		152		184
30-40	Male non-smokers	38397	74		133		186

	Female non-smokers	41455	74		145		205
	Male smokers	56808	95		133		167
	Female smokers	45420	103		150		191
	Male non-smokers	63108	67		132		190
	Female non-smokers	73723	61		139		208
	Male smokers	93921	92		134		172
40-50	Female smokers	89968	86		146		198
	Male non-smokers	43076	59		127		190
	Female non-smokers	53424	53		132		205
	Male smokers	66133	78		131		177
50-60	Female smokers	78393	62		139		206
	Male non-smokers	24424	59		121		182
	Female non-smokers	23448	52		124		196
	Male smokers	41919	61		123		181
60-70	Female smokers	42438	48		128		203
	Male non-smokers	18840	91		134		174
	Female non-smokers	12751	113		152		188
	Male smokers	29410	97		131		164
70>	Female smokers	10103	113		152		188

Table S3. The number of features, list of features, sample size before exclusion of outliers and sample size after exclusion of outliers for the three best-performing models.

Number of features	Features	Sample size before exclusion of outliers	Sample size after exclusion of outliers
**18**	Alanine Aminotransferase', 'Alkaline Phosphatase', 'Creatinine', 'Gender', 'Glucose Fasting', 'HDL Cholesterol', 'Hemoglobin', 'LDL Cholesterol', 'MCHC', 'Mean Corpuscular Volume', 'Platelet Count', 'RDW', 'Red Blood Count', 'Triglycerides' with four reconstructed for the part of samples: 'Bilirubin Total', 'Gamma-GT', 'Lactate Dehydrogenase', 'Urea'	**44491**	**39889**
**20**	Alanine Aminotransferase', 'Albumin', 'Alkaline Phosphatase', 'Creatinine', 'Gender', 'Glucose Fasting', 'HDL Cholesterol', 'Hemoglobin', 'LDL Cholesterol', 'MCHC', 'Mean Corpuscular Volume', 'Platelet Count', 'RDW', 'Red Blood Count', 'Triglycerides' with five reconstructed for the part of samples: 'Gamma-GT', 'Lactate Dehydrogenase', 'Urea', 'Protein Total', 'Calcium'	**20699**	**17816**

**23**	Alanine Aminotransferase', 'Albumin', 'Cholesterol', 'Creatinine', 'Gender', 'Glucose Fasting', 'HDL Cholesterol', 'Hematocrit', 'Hemoglobin', 'LDL Cholesterol', 'MCHC', 'Mean Corpuscular Volume', 'Platelet Count', 'Potassium', 'RDW', 'Red Blood Count', 'Sodium', 'Triglycerides' with five reconstructed for the part of samples: 'Bilirubin Total', 'Gamma-GT', 'Urea', 'Protein Total', 'Calcium	**19757**	**18607**

Table 4S

Age group	Group	Number of samples	Lower whisker	Lower hinge	Median	Upper hinge	Upper whisker	W Statistics	p-value
Age predictor trained on 18 features									
	Male non-smok	237	-0.415	-0.052	0.120	0.455	0.949	7954.5	1.51E-23
	Male smokers	233	-0.531	0.000	0.199	0.593	1.164	7954.5	1.51E-23
	Female non-sm	171	0.138	0.509	0.684	0.907	1.263	7565.5	3.20E-42
<30	Female smoker	225	0.096	0.500	0.716	0.911	1.284	7565.5	3.20E-42
	Male non-smok	604	-0.263	-0.044	0.037	0.183	0.406	78151.5	3.88E-56
	Male smokers	574	-0.322	-0.040	0.044	0.244	0.527	78151.5	3.88E-56
	Female non-sm	578	-0.193	0.188	0.385	0.585	0.981	72722	1.22E-66
30-40	Female smoker	582	-0.133	0.193	0.379	0.541	0.883	72722	1.22E-66
	Male non-smok	1149	-0.202	-0.060	0.000	0.089	0.231	327940.5	7.72E-24
	Male smokers	774	-0.162	-0.035	0.029	0.095	0.222	327940.5	7.72E-24
	Female non-sm	1054	-0.282	0.000	0.152	0.284	0.562	285564.5	1.56E-40
40-50	Female smoker	850	-0.322	-0.032	0.138	0.263	0.549	285564.5	1.56E-40
	Male non-smok	1703	-0.152	-0.052	0.000	0.052	0.152	1147059.5	3.03E-06
	Male smokers	1263	-0.158	-0.055	0.000	0.048	0.149	1147059.5	3.03E-06
	Female non-sm	1661	-0.295	-0.077	0.048	0.143	0.363	912629	1.59E-12
50-60	Female smoker	1527	-0.309	-0.103	0.000	0.107	0.317	912629	1.59E-12
	Male non-smok	1078	-0.215	-0.109	-0.042	0.000	0.107	715943	2.04E-08
	Male smokers	849	-0.237	-0.119	-0.047	0.000	0.112	715943	2.04E-08
	Female non-sm	1336	-0.445	-0.237	-0.123	-0.022	0.190	587079.5	1.25E-26
60-70	Female smoker	942	-0.430	-0.215	-0.096	0.000	0.213	587079.5	1.25E-26
	Male non-smok	452	-0.489	-0.255	-0.105	-0.020	0.115	102220.5	5.50E-17
	Male smokers	312	-0.464	-0.250	-0.115	-0.020	0.097	102220.5	5.50E-17
	Female non-sm	488	-0.614	-0.402	-0.285	-0.189	0.020	105732	7.35E-32
70>	Female smoker	310	-0.566	-0.342	-0.217	-0.117	0.097	105732	7.35E-32
Age predictor trained on 20 features									
	Male non-smok	86	-0.354	-0.120	-0.052	0.333	0.769	827	1.36E-15
	Male smokers	106	-0.485	-0.120	0.102	0.429	0.968	827	1.36E-15
	Female non-sm	70	0.054	0.544	0.758	1.051	1.541	982.5	9.83E-21
<30	Female smoker	90	0.000	0.451	0.706	0.948	1.333	982.5	9.83E-21
	Male non-smok	275	-0.204	-0.044	0.000	0.124	0.278	13407.5	5.19E-36
	Male smokers	237	-0.294	-0.078	0.000	0.152	0.382	13407.5	5.19E-36

	Female non-sm	287	-0.204	0.188	0.405	0.585	0.980	11625	3.37E-36
30-40	Female smoker:	250	-0.156	0.178	0.372	0.547	0.893	11625	3.37E-36
	Male non-smok	489	-0.131	-0.033	0.000	0.066	0.163	60303.5	2.06E-23
	Male smokers	327	-0.134	-0.034	0.000	0.069	0.167	60303.5	2.06E-23
	Female non-sm	508	-0.227	0.029	0.188	0.309	0.551	44368	3.77E-27
40-50	Female smoker:	388	-0.330	0.000	0.184	0.335	0.640	44368	3.77E-27
	Male non-smok	715	-0.105	-0.027	0.000	0.052	0.126	207082	1.51E-11
	Male smokers	596	-0.105	-0.028	0.000	0.050	0.126	207082	1.51E-11
	Female non-sm	741	-0.273	-0.052	0.053	0.173	0.390	167591	2.58E-11
50-60	Female smoker:	703	-0.246	-0.052	0.050	0.147	0.342	167591	2.58E-11
	Male non-smok	514	-0.202	-0.090	-0.022	0.022	0.133	134161	1.59E-02
	Male smokers	411	-0.205	-0.091	-0.021	0.023	0.135	134161	1.59E-02
	Female non-sm	519	-0.437	-0.215	-0.114	0.023	0.259	126969.5	1.23E-07
60-70	Female smoker:	475	-0.385	-0.170	-0.047	0.047	0.259	126969.5	1.23E-07
	Male non-smok	216	-0.455	-0.229	-0.078	0.000	0.136	20282	2.04E-12
	Male smokers	123	-0.336	-0.170	-0.040	0.000	0.136	20282	2.04E-12
	Female non-sm	232	-0.522	-0.346	-0.267	-0.169	0.000	19741.5	9.83E-14
70>	Female smoker:	120	-0.506	-0.310	-0.195	-0.103	0.060	19741.5	9.83E-14
Age predictor trained on 23 features									
	Male non-smok	105	-0.415	-0.158	0.000	0.379	0.898	1063.5	5.12E-17
	Male smokers	113	-0.485	-0.107	0.000	0.363	0.786	1063.5	5.12E-17
	Female non-sm	75	0.000	0.459	0.678	0.970	1.422	1726	1.58E-19
<30	Female smoker:	106	0.093	0.427	0.668	0.903	1.333	1726	1.58E-19
	Male non-smok	315	-0.271	-0.074	0.000	0.124	0.314	16475.5	2.92E-42
	Male smokers	255	-0.285	-0.074	0.000	0.138	0.348	16475.5	2.92E-42
	Female non-sm	301	-0.186	0.178	0.379	0.578	0.953	14039	9.63E-41
30-40	Female smoker:	304	-0.160	0.181	0.358	0.547	0.910	14039	9.63E-41
	Male non-smok	510	-0.126	-0.036	0.000	0.060	0.155	71883	2.45E-12
	Male smokers	358	-0.155	-0.064	0.000	0.035	0.128	71883	2.45E-12
	Female non-sm	490	-0.300	0.000	0.140	0.303	0.596	56341.5	6.81E-22
40-50	Female smoker:	403	-0.396	-0.064	0.111	0.274	0.574	56341.5	6.81E-22
	Male non-smok	770	-0.103	-0.027	0.000	0.051	0.128	226909.5	1.20E-03
	Male smokers	619	-0.128	-0.051	0.000	0.028	0.105	226909.5	1.20E-03
	Female non-sm	727	-0.316	-0.074	0.070	0.171	0.415	192038	4.52E-10
50-60	Female smoker:	693	-0.315	-0.100	0.025	0.121	0.338	192038	4.52E-10

	Male non-smok	531	-0.167	-0.073	-0.021	0.022	0.114	156208	1.27E-04
	Male smokers	432	-0.164	-0.073	-0.022	0.022	0.114	156208	1.27E-04
	Female non-sm	562	-0.451	-0.205	-0.072	0.043	0.239	133374.5	1.35E-05
60-70	Female smoker:	489	-0.415	-0.186	-0.066	0.042	0.259	133374.5	1.35E-05
	Male non-smok	210	-0.382	-0.193	-0.040	0.000	0.081	20501	1.52E-12
	Male smokers	161	-0.403	-0.193	-0.039	0.018	0.097	20501	1.52E-12
	Female non-sm	217	-0.690	-0.421	-0.282	-0.150	0.117	25454.5	6.70E-17
70>	Female smoker:	130	-0.565	-0.337	-0.209	-0.107	0.115	25454.5	6.70E-17

Table 5S

**Age predicting models (without a smoking status)**			
N of features	N of hidden layers	N of neurones	Optimizer Specification
23	3	1200, 1000, 500	EVE with 0.0001 learning rate
18	3	1000, 1000, 500	ADAM optimizer with 0.001 learning rate
20	3	1200, 1000, 500	EVE optimizer with 0.00001 learning rate
**Age predicting models (with smoking status)**			
24	3	1200, 1000, 500	EVE with 0.0001 learning rate
19	3	1000, 1000, 500	ADAM optimizer with 0.001 learning rate
21	3	1200, 1000, 500	EVE optimizer with 0.00001 learning rate.
**Classification models**			
23	3	1000, 1000, 500	ADAM optimizer with 0.0001 learning rate
18	3	1000, 1000, 500	EVE optimizer with 0.0001 learning rate.
20	3	1000, 1000, 500	EVE optimizer with 0.00001 learning rate
